# Neuronal MD2 induces long-term mental impairments in septic mice by facilitating necroptosis and apoptosis

**DOI:** 10.3389/fphar.2022.884821

**Published:** 2022-08-09

**Authors:** Zhongmin Fan, Hongwei Ma, Yi Li, You Wu, Jiajia Wang, Lize Xiong, Zongping Fang, Xijing Zhang

**Affiliations:** ^1^ Department of Critical Care Medicine and Department of Anesthesiology and Perioprative Medicine, Xijing Hospital, Fourth Military Medical University, Xi’an, China; ^2^ Translational Research Institute of Brain and Brain-Like Intelligence and Department of Anesthesiology and Perioperative Medicine, Shanghai Fourth People’s Hospital Affiliated to Tongji University School of Medicine, Shanghai, China

**Keywords:** sepsis-associated encephalopathy, mental impairments, neuronal death, myeloid differentiation factor 2, programmed cell death

## Abstract

Sepsis-associated encephalopathy (SAE) is a complication of sepsis with high morbidity rates. Long-lasting mental health issues in patients with SAE result in a substantial decrease in quality of life. However, its underlying mechanism is unclear, and effective treatments are not available. In the current study, we explored the role of apoptosis and necroptosis related to mental dysfunction in sepsis. In a mouse model of sepsis constructed by cecal ligation and puncture (CLP), altered behavior was detected by the open field, elevated-plus maze and forced swimming tests on the fourteenth day. Moreover, apoptosis- and necroptosis-associated proteins and morphological changes were examined in the hippocampus of septic mice. Long-lasting depression-like behaviors were detected in the CLP mice, as well as significant increases in neuronal apoptosis and necroptosis. Importantly, we found that apoptosis and necroptosis were related according to Ramsay’s rule in the brains of the septic mice. Inhibiting myeloid differentiation factor 2 (MD2), the crosstalk mediator of apoptosis and necroptosis, in neurons effectively reduced neuronal loss and alleviated depression-like behaviors in the septic mice. These results suggest that neuronal death in the hippocampus contributes to the mental impairments in SAE and that inhibiting neuronal MD2 is a new strategy for treating mental health issues in sepsis by inhibiting necroptosis and apoptosis.

## Introduction

Sepsis is a life-threatening organ dysfunction caused by a dysregulated response to infection (Singer et al., 2016), and is a major cause of death and disability (Napolitano, 2018). In septic patients, sepsis-associated encephalopathy (SAE) is a common complication that has a high mortality rate (Saito et al., 2021). As a diffuse cerebral dysfunction without direct central nervous system (CNS) infection, SAE results in different levels of consciousness, such as delirium, coma and acute mental dysfunction, in patients (Gofton and Young, 2012). Moreover, 10%–58% of SAE survivors were found to suffer from long-term depression (Streck et al., 2008; Calsavara et al., 2013). However, the underlying mechanism of long-lasting depression in patients with SAE is still unclear.

As a regulated cell death pathway, programmed cell death (PCD), including apoptosis and necroptosis, plays important roles in sepsis. Previous studies have extensively explored the effects of apoptosis in SAE (Wang et al., 2011; Bedirli et al., 2018; Zhang et al., 2021). However, whether necroptosis is involved in SAE is unclear. Therefore, elucidating the underlying mechanism of apoptosis and necroptosis in neuronal loss in SAE may lead to treatments for long-term depression in patients with this condition. Our previous study confirmed that myeloid differentiation factor 2 (MD2) is the crosstalk mediator of various PCDs and that inhibition of MD2 effectively reduced stroke injury (Fang et al., 2021). Nevertheless, the role of MD2 in the pathology of SAE has not been demonstrated.

In the current study, we hypothesized that neuronal apoptosis and necroptosis in the hippocampus contribute to long-term depression induced by SAE. Targeting the upstream molecular mechanism of apoptosis and necroptosis, MD2, may reduce neuronal death in the hippocampus and alleviate SAE. To confirm this hypothesis, we used a mouse model of sepsis constructed by cecal ligation and puncture (CLP) and evaluated behavioral changes and neuronal death. Furthermore, by using CaMKII-Cre; MD2^fl/fl^ mice, the role of MD2 in the regulation of apoptosis and necroptosis in the hippocampus was explored. The aim of this study was to clarify the effects of neuronal PCD in long-term depression induced by SAE, provide new insights into relieving sepsis-associated depression, and suggest an effective method for clinical treatment.

## Materials and methods

### Animals

Male C57/BL6 mice aged 8 weeks (20–25 g) were purchased from Charles River Co., Ltd. (China, Beijing). For generation of conditional MD2 knockout (cKO) mouse lines with deletion of exon 1, male homozygous MD2^fl/fl^ mice on the C57BL/6J genetic background were crossed with female MD2^fl/+^ mice carrying Cre. Heterozygous MD2^fl/+^ mice were generated by crossing female MD2 cassette (MD2 cassette/+) mice carrying a targeted MD2 allele on the C57BL/6J genetic background with male protamine-Flp mice. CaMKII-Cre (#5359) mice with a C57BL/6J genetic background were obtained from The Jackson Laboratory. PCR genotyping of CaMKII cKO mice was performed using the following set of oligonucleotide primers: floxed allele, forward, 5′-TCT​CAG​TAC​TTC​GGA​GGC​AGG​ATG​A-3′, reverse, 5′-TAC​CCT​CCT​TTC​ACT​CCC​TCG​TTC​C-3'; Cre allele, forward, 5′-GGT​TCT​CCG​TTT​GCA​CTC​AGG​A-3′, reverse, 5′-CCTGTTGTTCA GCTTGCACCAG-3'. Mice were housed under standard conditions (22 ± 2°C, humidity 50%, and 12 h light-dark cycle from 8 a.m. to 8 p.m.) and had free access to food and water. All animal procedures were carried out in accordance with the guidelines for the Care and Use of Laboratory Animals of Fourth Military Medical University (and the number of this approval 20200515).

### Antibodies and reagents

The following primary antibodies were used: rabbit anti-RIPK1/RIP1 (phospho Ser166) antibody (AGR66476, Arigo), rabbit anti-MLKL (phospho S345) antibody (ab196436, Abcam), rabbit anti-caspase-3 antibody (#9662, CST), rabbit anti-cleaved caspase-3 (Asp175) antibody (#9661, CST), rabbit anti-β-actin (13E5) antibody (#4970, CST), rabbit anti-MD2 antibody (No. GTX85517, GeneTex), and mouse anti-NeuN antibody [1B7] (ab104224, Abcam). The secondary fluorophore-bound IgGs (Alexa Fluor series) for immunofluorescence (IF) included donkey anti-rabbit IgG H&L (Alexa Fluor^®^ 594) (ab150064, Abcam) and donkey anti-mouse IgG H&L (Alexa Fluor^®^ 488) (ab150105, Abcam). Inhibitors, including Z-VAD-FMK [ab120487, Abcam, 5 μg/μl, intracerebroventricular (icv) administration], necrostatin-1 (Selleck, 3 μg/μl, icv), protease inhibitor cocktail (No. 16B140042, Biotool) and phosphatase inhibitor cocktail (No. B15002, Biotool) were used. Tat-fused peptides were custom-synthesized by the Peptide Synthesis and Purification Core Facility at GL Biochem. NeuroTrace™ Nissl 500/525 green fluorescent stains were purchased from Invitrogen.

### Cecal ligation and puncture

The mice were fasted for 12 h but had free access to water before the surgical procedure. Then, anesthesia was induced by inhalation of 2% isoflurane through a face mask, and anesthesia was maintained with 1.5% isoflurane. Under sterile conditions, the cecum was ligated with 4–0 silk at the midpoint and punctured once with a 22-gauge needle. Then, the cecum was gently squeezed until a single droplet of fecal material came out of the puncture site. Next, the abdominal cavity was closed in two layers, followed by fluid resuscitation (preheated saline, 20 ml/kg), and the animal was returned to its cage. In the sham group, except for ligation and perforation, the rest of the steps were the same as those in the CLP model group. All animals received care with suitable analgesia according to institutional guidelines.

### Peptide design

According to the high affinity between the cold-inducible RNA binding protein CIRP and MD2 (Qiang et al., 2013), we synthesized a blocking peptide to mimic the 106–125 domain of CIRP and link the trans-trans-activating (Tat) transmembrane functional domain (YGRKKRRQRRR) to form Tat-CIRP (YGRKKRRQRRR-GRGFSRGGGD RGYGG), which promotes penetration of the BBB. For other details, see our previous study (Fang et al., 2021).

### Western blot analysis

Western blot analysis was carried out on the protein samples from the hippocampus. The mice were sacrificed, and the hippocampus was collected and stored at −80°C. Fresh hippocampal tissues were weighed and homogenized in lysis buffer containing protease and phosphatase inhibitors. After lysis on ice for 20 min, the homogenates were centrifuged at 12,000 rpm for 10 min at 4°C. Then, the supernatant was collected, and 10 µl was used for BCA protein quantification. The residual supernatant was denatured with 4×loading buffer at 100°C for 10 min and stored in a −20°C freezer for later use. Subsequently, 10 µl of the prepared sample was separated by SDS-PAGE and transferred to a PVDF membrane under a voltage of 100 V for 90 min. After the membrane was blocked with 50 g/l BSA or 5% nonfat milk for 2 h, it was incubated with primary antibody overnight in a refrigerator at 4°C followed by incubation with HRP-labeled IgG for 2 h at room temperature. After thorough washing with TBST, the Bio-Rad gel imaging system was used for chemical development.

### Nissl staining

For Nissl staining, after deparaffinization and rehydration, the slices were immersed in 100% ethanol for approximately 5 min, 95% ethanol for 30 s, and 70% ethanol for 30 s. The samples were then washed in PBS three times, immersed in 1% Nissl for 8 min, and washed with PBS three times. Next, the samples were immersed in 70% ethanol for 2 min, 80% ethanol for 2 min, 95% ethanol for 2 min, 100% ethanol for 2 min (twice) and xylene for 2 min (twice). Finally, the slices were sealed by neutral gum. For TeuroTrace™ fluorescent Nissl staining, cryosections (10 μm) were prepared with standard protocols. Then, the samples were rehydrated in PBS for 40 min and washed twice for 10 min in PBS plus 0.1% Triton X-100. Next, the sections were covered with NeuroTrace™ stain (1:2,000) for at least 20 min. After thorough washing, DAPI-containing mounting tablets were used for mounting.

### Immunofluorescence staining

Mice were deeply anesthetized with 2% pentobarbital sodium (20 mg/kg, i.p.) and was rapidly perfused 50 ml of pre-cooled saline through the heart, followed by slow perfusion with 50 ml of 4% paraformaldehyde. The brain was taken out and fixed for 2 h in 4% paraformaldehyde. Then they were dehydrated with 20% and 30% sucrose solutions in sequence until the brain sank to the bottom. And they were made into frozen tissue section in 10 μm. The brain slices were washed 3 times with PBS for 10 min each time. After washing, the slices were blocked with donkey serum and 0.05% Triton X-100 for 2 h at room temperature, and were incubated with primary antibodies overnight at 4°C. After washing thoroughly, secondary antibody was added to the slices in a dark box for 2 h. After thorough washing, DAPI-containing mounting tablets were used to mount the slices. Then, they were placed in a confocal microscope.

### Stereotactic injection

Tat-CIRP or inhibitors were injected into lateral ventricles through stereotactic technology. Mice were anesthetized with 2% pentobarbital sodium (20 mg/kg, i.p.) and fixed in a stereotaxic device. Then, their eyes were covered by erythromycin ointment to prevent injury caused by long-term exposure. After a series of procedures, we identified the coordinates of the lateral ventricles (AP = −0.5 mm, ML = +1.0 mm, DV = −1.4 mm) and injected relevant drugs at a total volume of 1 μl.

### Open field test

The OFT is one of the most commonly used tests to assess anxiety-like behaviors in rodents and allows animals to freely explore open areas surrounded by walls. Under normal conditions, the animals show thigmotaxis (tend to avoid the central region and stay at the peripheral region), and increased or decreased center activity is associated with anxiolysis or anxiogenesis (Kuniishi et al., 2017). First, the mice were handled starting 14 days prior to the experiment to allow acclimatization to the investigator. Additionally, the mice were placed in the testing room 1 h before testing to acclimatize to the environment. The mice were placed in an apparatus consisting of a 40 cm × 40 cm × 40 cm polyvinyl chloride (PVC) box, and a camera was used to monitor their movements. All the mice were placed in the same starting position (the corner) of the PVC box at the start, and allowed to explore the test area for 5 min. During testing, the investigator left the room. The tests were all carried out between 6:30 p.m. and 10:30 p.m. After each test, the mice were returned to the cage, and the bottom and sidewalls of the box were cleaned with 75% alcohol. The next step did not start until the box was dry and there was no smell. Mouse movements and activity were recorded and analyzed by the Anymaze system.

### Elevated plus maze

An EPM is one of the traditional tests to evaluate anxiety-like behavior. In this test, four elevated arms radiate from a central platform in which two opposing arms are walled and the other two opposing arms are opened. A camera was placed on a stand above the arena. Before the test, the mice were allowed to acclimatize to the facility for 7 days. In addition, the mice were moved to the behavioral testing room 1 h before the test. During testing, the mice were placed at the intersection of the open and closed arms of the elevated plus maze and allowed to freely explore the maze for a total of 5 min. During testing, the investigator left the room. Mice tended to avoid open or elevated places, which was counterbalanced by their innate curiosity to explore areas that are new to them. More time in the open arms was associated with less anxiety, and more time in the closed arms was related to anxiety. Additionally, the tests were all carried out between 6:30 p.m. and 10:30 p.m. After each test, the mice were returned to the cage, and the arms were cleaned with 75% alcohol. The next step did not start until the device was dry and there was no smell. Mouse movements and activity were recorded and analyzed by the Anymaze system.

### Forced swimming test

Similar to the other tests, the mice were given 7 days to acclimate to the testing facility and the investigator before the test. In addition, the mice were moved to the behavioral testing room 1 h before the test. During the test, mice were subjected to an 8-min swim session including 2 min in the adaptation stage and 5 min in the test stage in clear plexiglass cylinders (30 cm height × 20 cm diameter) filled with 17 cm of water (23 ± 2 °C). The water was changed every three mice. The test was performed between 6:30 p.m. and 10:30 p.m. under normal light conditions. The tests were recorded by a digital video camera, and the investigator left the room during the test. The immobility time, which was defined as passive floating with no additional activity other than that necessary to keep the animal’s head above the water was recorded. After each test, the mice were dried off and placed in an incubator until they recovered.

### Statistical analysis

GraphPad Prism 8.0.2 was used to conduct the statistical analysis. All values except for the murine sepsis score (MSS) and the weights are presented as the mean ± SEM and were analyzed by using two-tailed Student’s *t* test (comparisons of two groups) or one-way analysis of variance (ANOVA) followed by Dunnett’s or Tukey’s post hoc test (comparisons of more than two groups). Survival curves and comparisons among curves were assessed through the Mantel-Cox log-rank test. A *p* value less than 0.05 was defined as significant.

## Results

### Sepsis induced long-term mental impairments in mice that underwent cecal ligation and puncture

First, the severity of sepsis in the mice was evaluated by MSS, following previous research (Shrum et al., 2014). In CLP mice, there was a positive correlation between the MSS and the level of serum IL-1β (Supplementary Figure S1C), indicating that the MSS reflected the severity of sepsis induced by CLP in these mice. The MSS significantly increased during the first 3 days and peaked on the third day. Then, the MSS gradually decreased in the following days (Supplementary Figure S1A), indicating the alleviation of sepsis. Similarly, the weight of the CLP-induced mice declined after the surgery and reached a minimum on the third day (Supplementary Figure S1B). Total distance (TD) as an indicator of OFT is often used to evaluate the general health of animals. Our results showed no difference in TD between the sham-treated and CLP-induced mice at 14 days after sepsis (Supplementary Figure S1F), which indicated that evaluating anxiety and depressive behaviors at this time point was reasonable. Then, the relationship between the MSS and outcome was analyzed. Through logistic regression, a significant relationship between scores on the first and the third day and the final mortality was found (Supplementary Table S1). Then, we established a group trajectory development model according to the MSS and final outcomes. The animals were divided into three groups according to their results. The first group showed a low probability of death with MSS<5 all times, and the third group presented high mortality in the first 3 days with MSS>10at the beginning and gradually increasing with time. The second group showed a balance between survival and death, with an inflection point at MSS≥5 on the third day relative to the first group (Supplementary Figure S1E; Supplementary Table S2). Therefore, we selected mice with MSS≥5 on the third day after CLP for subsequent behavioral experiments.

**FIGURE 1:**
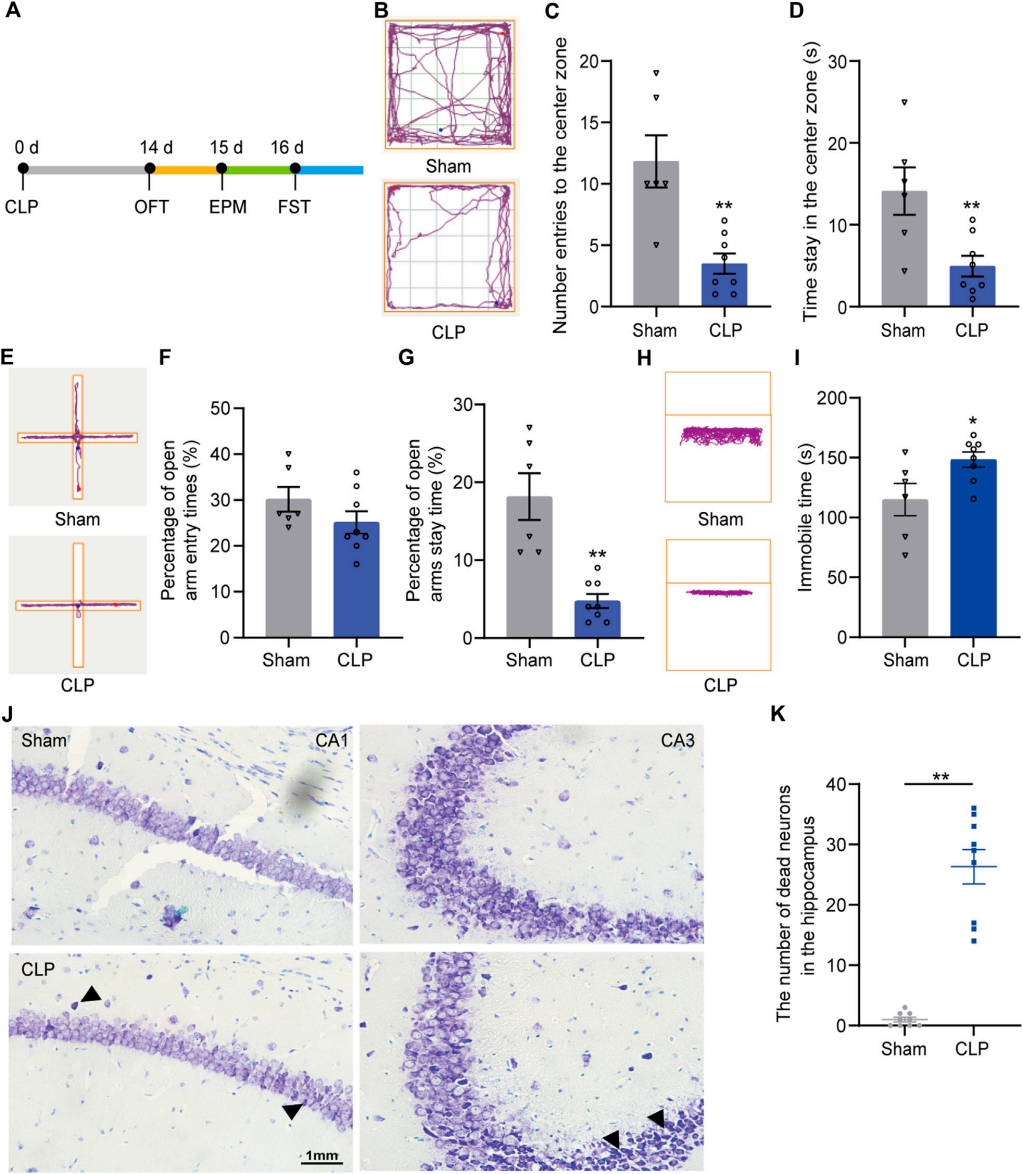
Septic mice showed long-term depression-like behaviors followed by loss of neurons in the hippocampus. **(A)**. An outline of the experimental procedure for mice with CLP surgery and behavioral tests. **(B)**. Typical tracking chart of the OPF between the sham and CLP. **(C)**. The differences between the sham and CLP in the number of entries to the center zone. **(D)**. The differences between the sham and CLP in time spent in the center zone in the OFT (n sham = 6, n CLP = 8). **p < 0.01 vs. the sham. Data are shown as the mean ± SEM. **(E)**. Typical tracking chart of the EPM between the sham and CLP. **(F)**. In the EPM, percentage of entries to the open arms. **(G)**. Percentage of time spent in the open arms (n sham = 6, n CLP = 8). **p < 0.01 vs. the sham group. Data are shown as the mean ± SEM. **(H)**. Typical tracking chart of the FST between the sham and CLP. **(I)**. Total immobility time during 5 min in the FST (n sham = 6, n CLP = 8). *p < 0.05 vs. the sham. Data are shown as the mean ± SEM. **(J)**. Nissl staining in the hippocampus of the sham and CLP 14 d after CLP. **(K)**. The number of dead neurons in the hippocampus between the sham and CLP. **p < 0.01 vs. the sham. Data are shown as the mean ± SEM (*n* = 9).

Next, a battery of behavioral tests was performed at 14 days after CLP to determine whether the septic mice present depression-like behaviors (Figure 1A). The OFT (Figure 1B), EPM (Figure 1E) and FST (Figure 1H) were performed. In the OFT, the number of entries to the center zone (Figure 1C) and the time spent in the center zone (Figure 1D) were decreased in the CLP group compared with the sham group. Similarly, the percentage of time spent in the open arm of the septic mice was significantly reduced in the EPM (Figure 1G). However, the animals presented no difference in the percentage of entry in the open arm (Figure 1F). The immobility time of the septic mice was increased in the FST (Figure 1I). The above results indicated that CLP induced long-term mental impairments in mice.

Then, we explored the possible mechanism of sustaining depression-like behaviors induced by sepsis. Pathological staining showed substantial neuronal death in the hippocampus of the septic mice (Figures 1J,K). These findings indicate the probable mechanism of hippocampal neuronal death underlying the long-lasting depression-like behaviors of the septic mice.

### Both apoptosis and necroptosis in hippocampal neurons were increased in the septic mice

**FIGURE 2:**
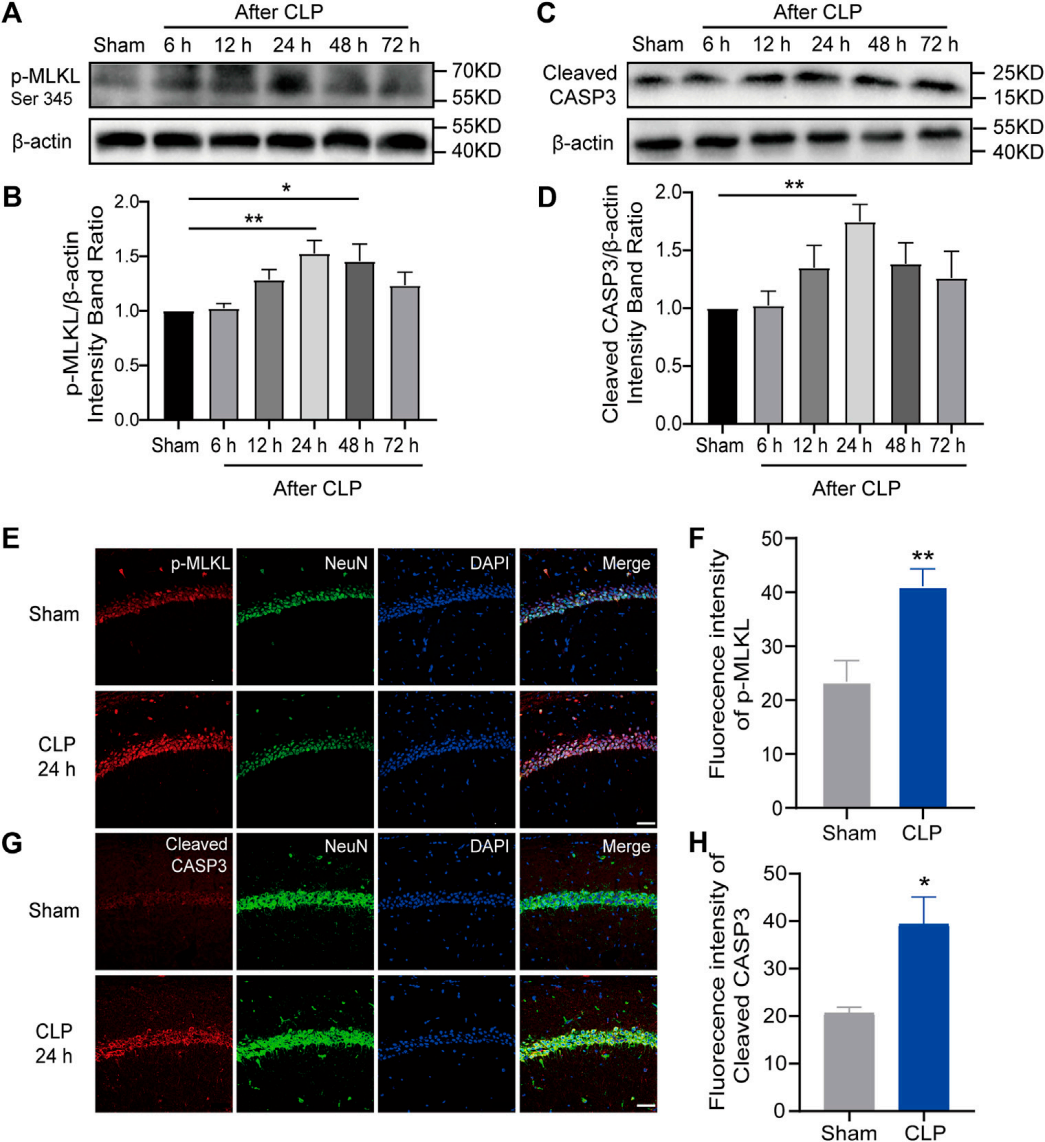
Apoptosis and necroptosis were increased in the hippocampus of mice with sepsis. **(A)**. Representative Western blot of p-MLKL. **(B)**. Quantitative evaluation of p-MLKL expression after CLP at the time point. **p < 0.01 vs. the sham. Data are shown as the mean ± SEM (*n* = 9). **(C)**. Representative Western blot of cleaved caspase-3. **(D)**. Quantitative evaluation of cleaved caspase-3 expression after CLP at the time point. **p < 0.01 vs. the sham. Data are shown as the mean ± SEM (*n* = 9). **(E)**. IF staining of p-MLKL 24 h after CLP (bar = 40 μm). **(F)**. Analysis of the intensity of p-MLKL between the sham and CLP. **p < 0.01 vs. the sham. Data are shown as the mean ± SEM (n = 9). **(G)**. IF staining of cleaved caspase-3 24 h after CLP (bar = 40 μm). **(H)**. Analysis of the intensity of cleaved caspase-3 between the sham and CLP groups. *p < 0.05 vs. the sham. Data are shown as the mean ± SEM (*n* = 9).

Apoptosis and necroptosis are two major PCDs that participate in the pathological processes of sepsis. To investigate their roles in SAE in septic mice, apoptosis-associated proteins and necroptosis-associated proteins in the hippocampus were detected. Our Western blot results indicated a marked increase in the amounts of p-Ripk1, p-MLKL (necroptosis markers) and cleaved caspase-3 (apoptosis markers) in the hippocampus during the first 3 days and a peak at 24 h after CLP surgery (Figures 2A–D; Supplementary Figure S2A–D). Moreover, the IF results demonstrated increased apoptosis and necroptosis in hippocampal neurons at 24 h after CLP (Figures 2E–H; Supplementary Figure S2E–H). These results revealed that apoptosis and necroptosis were increased in hippocampal neurons, which may mediate the pathological process of SAE.

### Apoptosis and necroptosis were related according to Ramsay’s rule in the brains of the septic mice

Next, we tested whether suppression of apoptosis or necroptosis in the hippocampus can alleviate the behavioral changes induced by sepsis. Necrostatin-1 (Nec-1) [a specific inhibitor of necroptosis (Cao and Mu, 2021)] and benzyloxycarbonyl-Val-Ala-Asp-fluoromethyl ketone (Z-VAD-FMK) [a specific inhibitor of apoptosis (Chang et al., 2020)] were administered intracerebroventricularly to mice immediately after CLP surgery. Then, their behaviors were observed 14 days after CLP (Supplementary Figure S3A, B). Surprisingly, there were no differences among the groups in survival rate or mental disorder-associated behaviors (Supplementary Figure S3C–H). Moreover, there was no significant improvement in the MSS or weight or reduction in neuronal loss under inhibitor treatment (Supplementary Figure S4, S5). These results indicated that inhibiting apoptosis or necroptosis alone could not rescue the depression of the septic mice.

**FIGURE 3:**
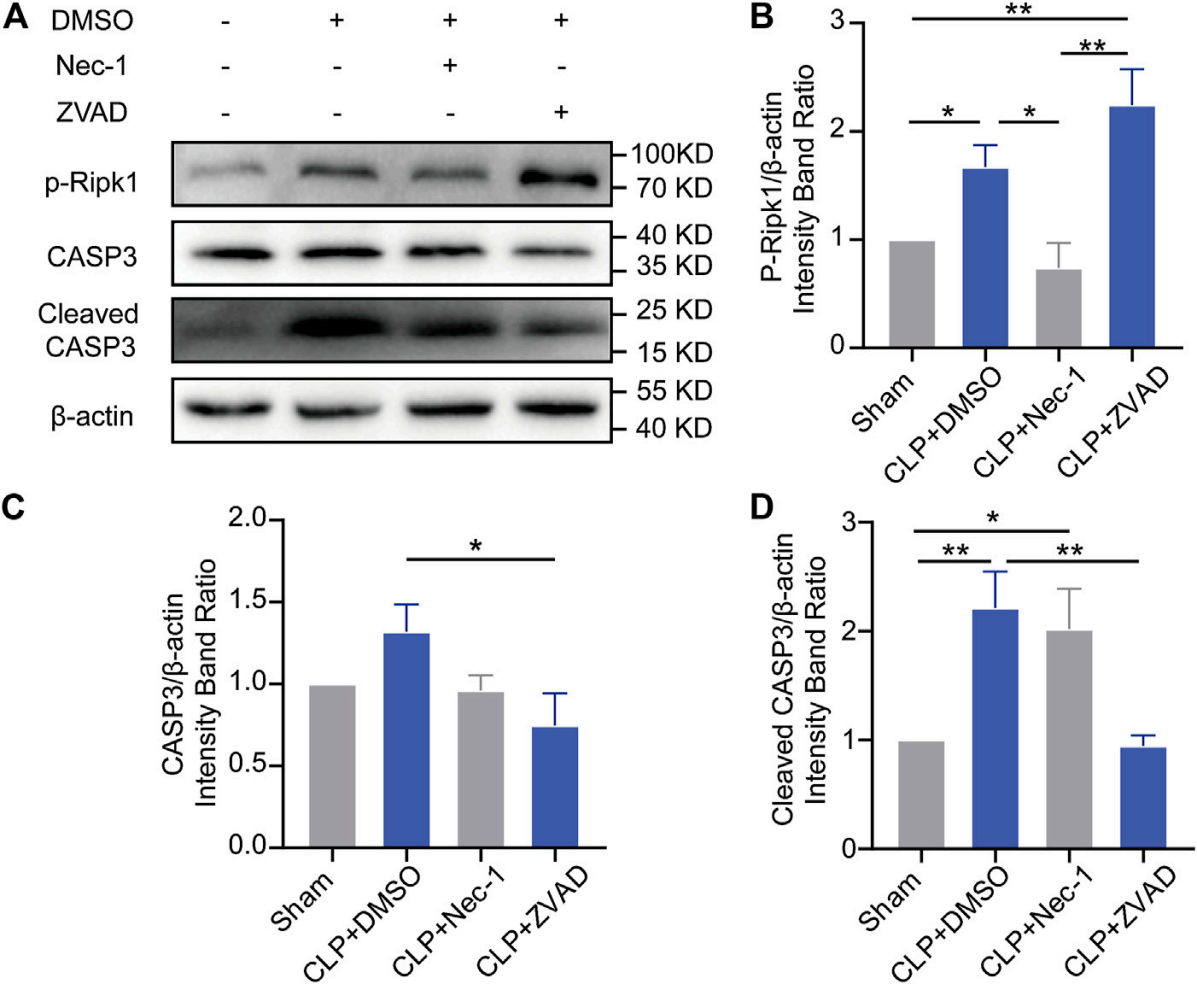
Apoptosis and necroptosis showed Ramsay’s rule in the brains of septic mice. **(A)**. Representative Western blots of p-Ripk1, caspase-3 and cleaved caspase-3. **(B)**. Quantitative evaluation of p-Ripk1 expression with different treatments. **(C)**. Quantitative evaluation of caspase-3 expression with different treatments. **(D)**. Quantitative evaluation of cleaved caspase-3 expression with different treatments. *p < 0.05, **p < 0.01. Data are shown as the mean ± SEM (*n* = 4).

Then, we explored the reasons why inhibition of apoptosis or necroptosis failed to alleviate depression in the septic mice. Our results showed that Nec-1 treatment facilitated the expression of cleaved caspase-3 and that the administration of Z-VAD-FMK accelerated the phosphorylation of Ripk1 (Figures 3A–D), which indicated a relationship according to Ramsay’s rule between apoptosis and necroptosis. However, synergy using Nec-1 and Z-VAD-FMK increased the death of the septic mice (Supplementary Figure S3C), which may be attributed to the superimposed toxicity of inhibitors and solvent.

### MD2 synchronously mediated apoptosis and necroptosis, and excitatory neuronal MD2 deletion relieved depression-like behaviors in the septic mice

**FIGURE 4:**
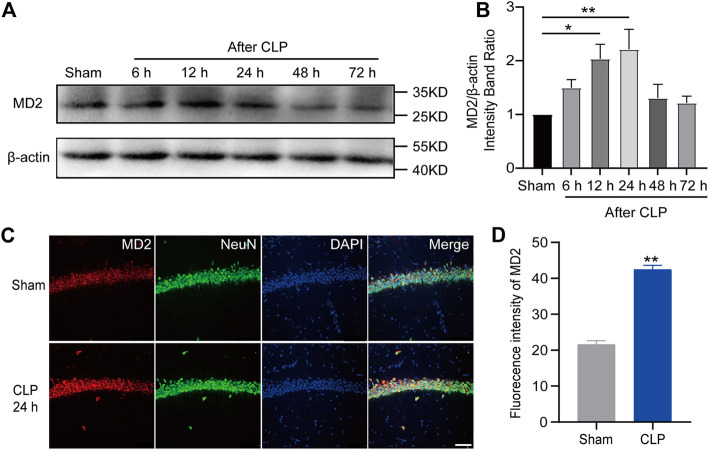
MD2 was increased in hippocampal neurons in septic mice. **(A)**. Representative Western blot of MD2. **(B)**. Quantitative evaluation of MD2 expression after CLP at the time point. **p < 0.01 vs. the sham. Data are shown as the mean ± SEM (*n* = 9). **(C)**. IF staining of MD2 24 h after CLP (bar = 40 μm). **(D)**. Analysis of the intensity of MD2 between the sham and CLP. **p < 0.01 vs. the sham. Data are shown as the mean ± SEM (*n* = 9).

**FIGURE 5:**
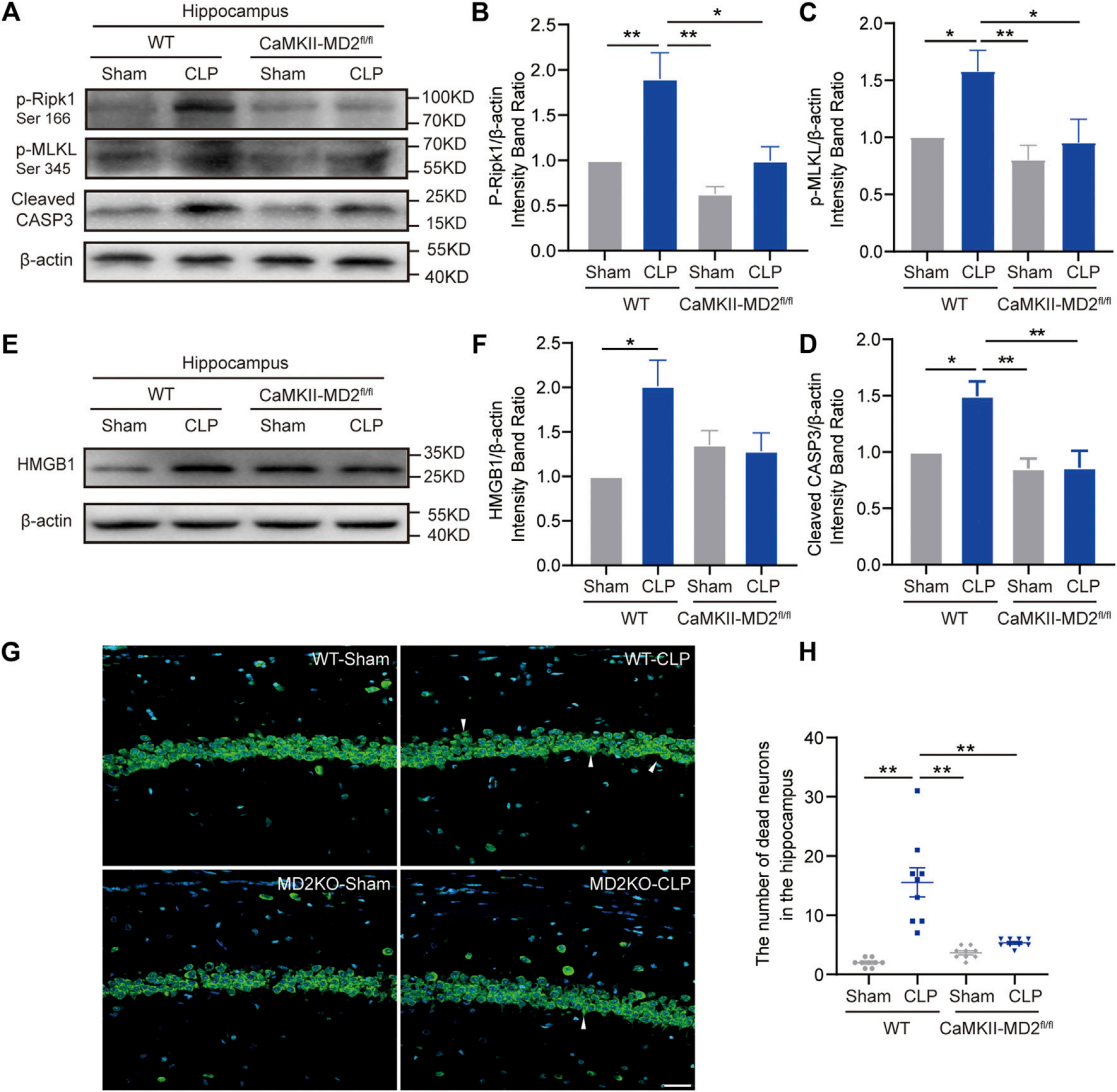
MD2 synchronously regulated apoptosis and necroptosis in hippocampal neurons, and inhibition of MD2 reduced cell death. **(A)**. Representative Western blots of p-Ripk1, p-MLKL and cleaved caspase-3 in different groups. **(B)**. Quantitative evaluation of p-Ripk1 expression. **(C)**. Quantitative evaluation of p-MLKL expression. **(D)**. Quantitative evaluation of cleaved caspase-3 expression 24 h after CLP in different groups. *p < 0.05, **p < 0.01 vs*.* the CLP-WT group. Data are shown as the mean ± SEM (*n* = 8). **(E)**. Representative Western blot of HMGB1 24 h after CLP. **(F)**. Quantitative evaluation of HMGB1 24 h after CLP. *p < 0.05. Data are shown as the mean ± SEM (n = 8). **(G)**. NeuroTrace™ Nissl staining in the hippocampus at 14 days of CLP (bar = 30 μm). **(H)**. The number of dead neurons in the hippocampus among different groups. **p < 0.01. Data are shown as the mean ± SEM (*n* = 9).

**FIGURE 6:**
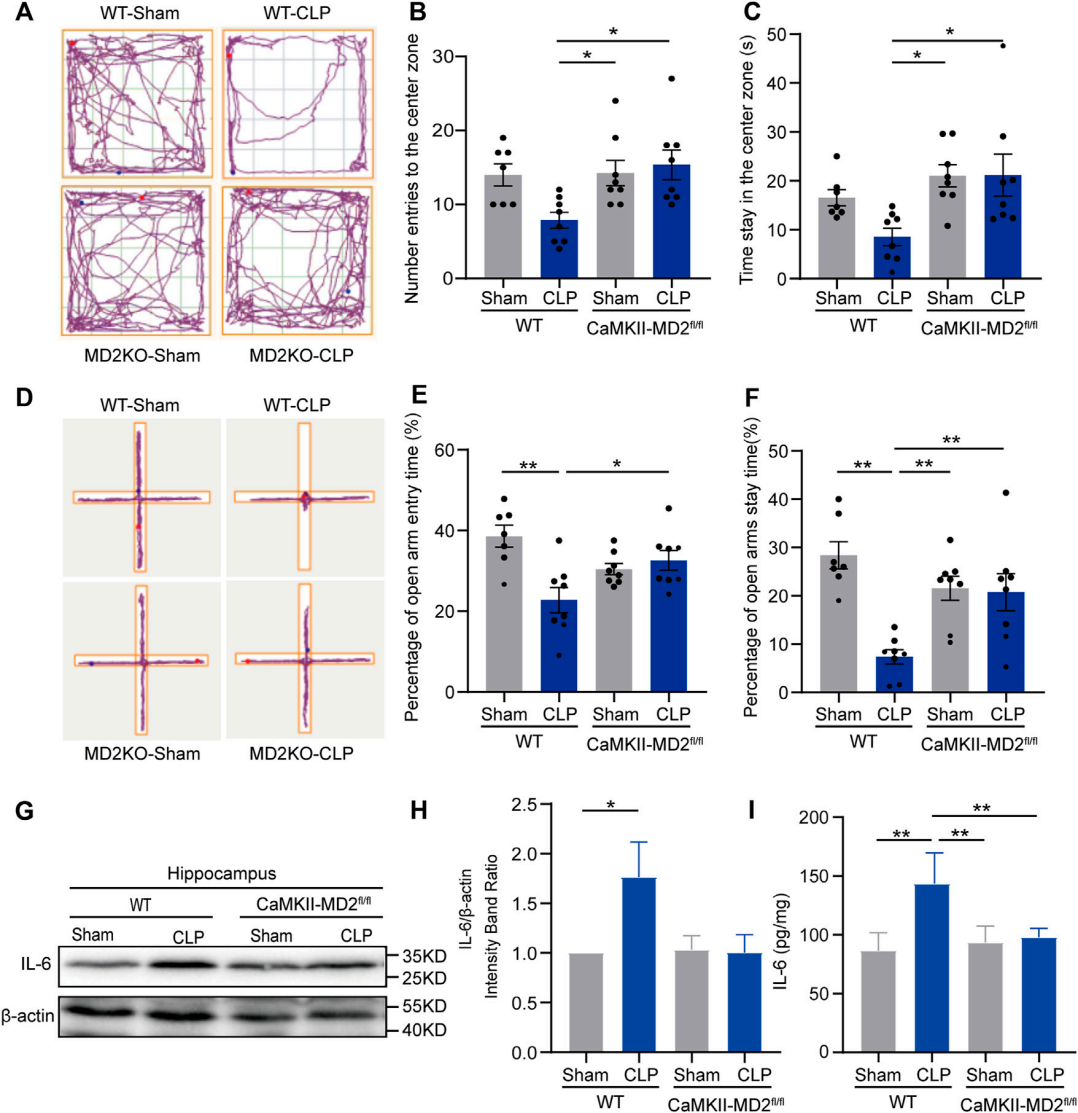
Depression-like behaviors were reduced in the CaMKII-MD2^fl/fl^ mice at 14 d after CLP. **(A)**. Typical tracking chart of the OPF between the CaMKII-MD2^fl/fl^ mice and the WT mice. **(B)**. The differences between the WT and CaMKII-MD2^fl/fl^ groups in number of entries to the center zone. **(C)**. The differences in time spent in the center zone in the OFT (n sham-WT = 7, others *n* = 8). *p < 0.05. Data are shown as the mean ± SEM. **(D)**. Typical tracking chart of the EPM between the CaMKII-MD2^fl/fl^ mice and the WT mice. **(E)**. The percentage of entries to the open arms. **(F)**. The percentage of time spent in the open arms (n sham-WT = 7, others *n* = 8). *p < 0.05, **p < 0.01. Data are shown as the mean ± SEM. **(G)**. Representative Western blots of IL-6 24 h after CLP. **(H)**. Quantitative evaluation of IL-6 in the hippocampus. *p < 0.05. Data are shown as the mean ± SEM (n = 8). **(I)**. ELISA analysis of IL-6 in the hippocampus among different groups. **p < 0.01. Data are shown as the mean ± SEM (*n* = 8).

Given the current unsatisfactory treatments, we attempted to identify a target that affects both apoptosis and necroptosis. MD2 is an essential cofactor protein of TLR4 that participates in the inflammatory response and is a key mediator of apoptosis and necroptosis in stroke models (Fang et al., 2021). Here, we explored the expression of MD2 in neurons in the hippocampus of the septic mice. A prominent increase in MD2 expression was detected at 24 h after CLP surgery (Figures 4A–D). To further confirm the crucial role of MD2 in the pathology of SAE, we constructed transgenic mice that with specific MD2 knockout in excitatory neurons (see our previous research for details (Fang et al., 2021)). The expression of apoptosis- and necroptosis-associated proteins was reduced in the hippocampus of the CaMKII-MD2^fl/fl^ mice compared with their littermates (Figures 5A–D). Moreover, the amount of HMGB1, a typical damage-associated molecular pattern (DAMP) molecule, was decreased (Figures 5E,F). Accordingly, less cell death was observed in the hippocampus of the CaMKII-MD2^fl/fl^ mice than the control mice (Figures 5G,H). Then, we tested proinflammatory factors in the hippocampus of the CaMKII-MD2^fl/fl^ mice. The expression of IL-6 was decreased in the CaMKII-MD2^fl/fl^ mice (Figures 6G–I).

Then, a series of behavioral experiments were performed (Supplementary Figure S6A), revealing that the CaMKII-MD2^fl/fl^ mice exhibited fewer behavioral changes 14 days after CLP (Figures 6A–F). Moreover, the MSS and weight loss of the CaMKII-MD2^fl/fl^ mice with sepsis were mitigated compared with those of their littermates (Supplementary Figure S6C, D).

### Local and systemic administration of TAT-CIRP alleviated depression-like behaviors and neuronal death in the septic mice

**FIGURE 7:**
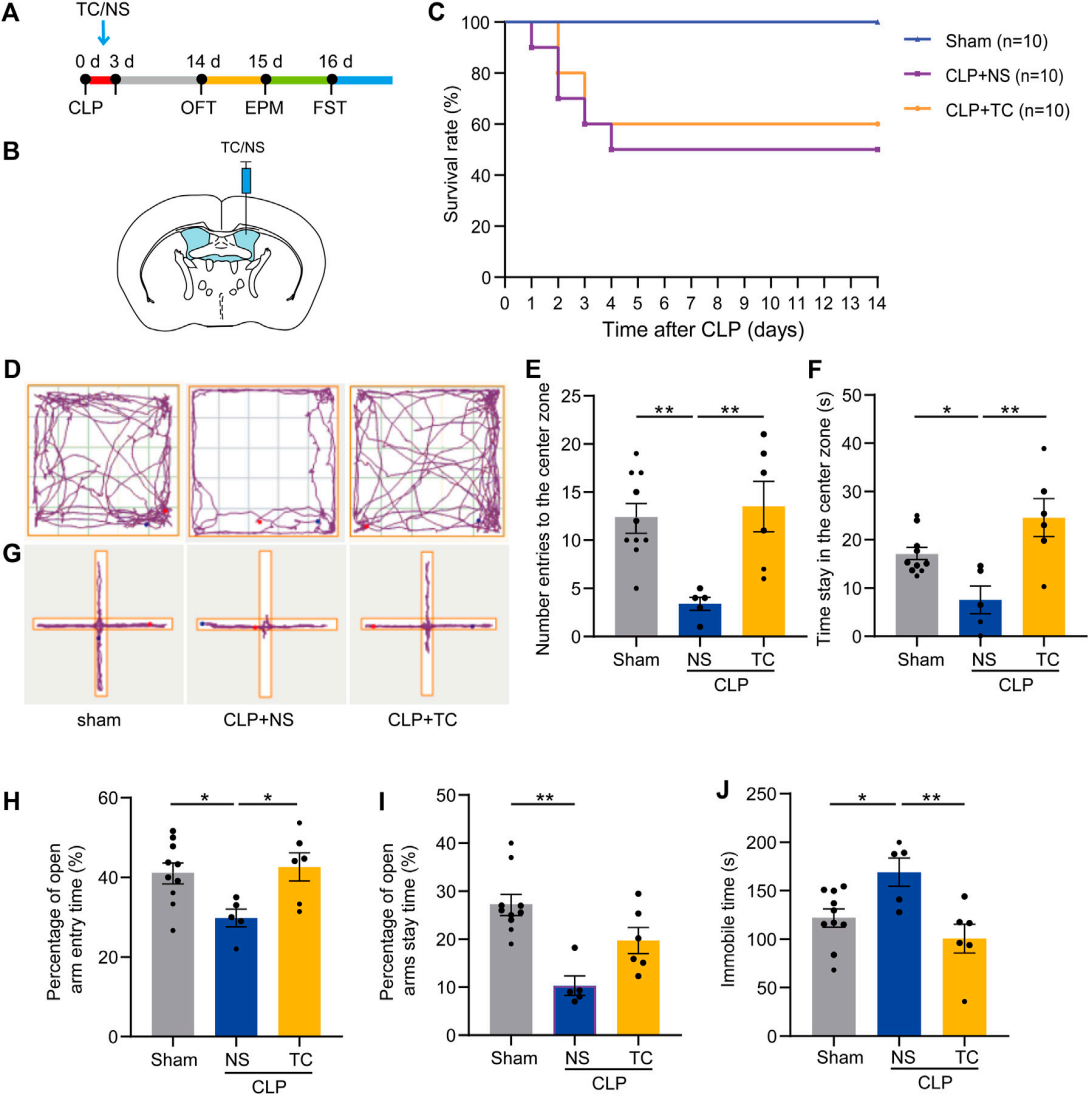
TAT-CIRP alleviated depression-like behaviors in mice with sepsis. **(A)**. An outline of the experimental procedure for TC injection and behavioral tests of mice with sepsis. **(B)**. Schematic configuration of icv injection. **(C)**. Survival curve for TC icv injection. 5of 10 mice in the CLP + NS group and 6 of 10 mice in the CLP + TC group survived by day 14. **(D)**. The representative tracking chart of the OFT. **(E)**. The effects of different treatments on number of entries to the center zone. **(F)**. The time spent in the center zone in the OFT (n sham = 9, n CLP + NS = 6, n CLP + TC = 6). *p < 0.05, **p < 0.01. Data are shown as the mean ± SEM. **(G)**. The representative tracking chart of the EPM. **(H)**. In the EPM, percentage of entries to the open arms. **(I)**. The percentage of time spent in the open arms (n sham = 9, n CLP + NS = 6, n CLP + TC = 6). *p < 0.05, **p < 0.01. Data are shown as the mean ± SEM. **(J)**. Total immobility time over 5 min in the FST among different groups (n sham = 9, n CLP + NS = 6, n CLP + TC = 6). *p < 0.05, **p < 0.01. Data are shown as the mean ± SEM.

**FIGURE 8:**
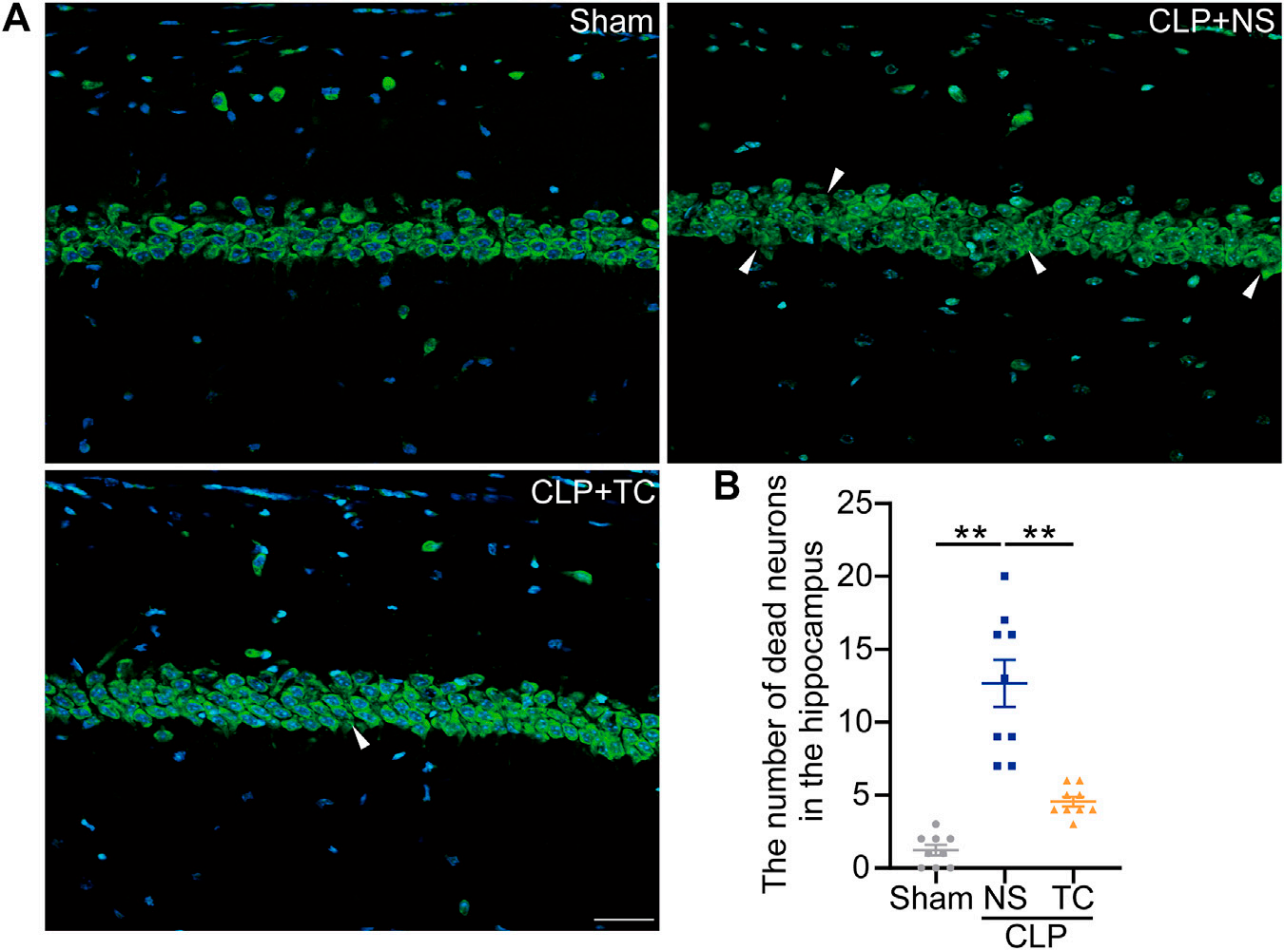
Neuronal loss in the hippocampus was reduced in the TAT-CIRP-treated septic mice. (**A)**. NeuroTrace™ Nissl staining in the hippocampus at 14 days after CLP (bar = 30 μm). **(B)**. The number of dead neurons in the hippocampus among different groups. *p < 0.05, **p < 0.01. Data are shown as the mean ± SEM (*n* = 9).

After demonstrating the critical role of MD2 in SAE, we tried to find a potential clinical treatment for SAE. In our previous study, we designed a short peptide to disturb the function of MD2 (Fang et al., 2021). To confirm its utility in the sepsis model, TC was administered *via* icv injection into the mice during the first 3 days after CLP, and the behaviors were tested on the fourteenth day after CLP (Figures 7A,B). The TC administration group showed fewer behavioral changes than the CLP group (Figures 7D–J). The MSS of the TC group was lower than that of the CLP group without any other treatment (Supplementary Figure S7A). The weight of the TC group declined quickly during the period of drug delivery; however, the speed and degree of weight recovery were better than those of the saline groups (Supplementary Figure S7B). There was only a trend of improved survival in the TC-treated group, which was treated with icv administration (Figure 7C). Neuronal loss in the septic mice was alleviated with TC (Figures 8A,B). These results show that TC has good application prospects for treating SAE. Then, the mice were systematically administered TC within 3 days after CLP with a dosage of 50 mg/kg. The survival rate, MSS, and weight of mice were recorded for 14 days after CLP. Behavioral changes were tested on the fourteenth day after CLP. The survival rate of CLP + TC group was increased compared with the CLP group (Supplementary Figure S8A). In the CLP + TC group, improvements in the MSS, and weight of mice were also accelerated (Supplementary Figure S8B–C). Furthermore, the percentage of open arm entries and durations in the open arms were increased in the CLP + TC group compared with the CLP group (Supplementary Figure S8D). These results indicate that TC facilitates recovery from sepsis.

## Discussion

It has been reported that more than 19 million septic patients each year (Prescott and Angus, 2018). As one of the major manifestations of SAE, long-term mental health issues affect as many as 58% of surviving septic patients (Prescott and Angus, 2018; Calsavara et al., 2021), leading to poor quality of life and a heavy economic burden. However, the concrete mechanism of long-lasting mental disorders in septic patients is not clear. As a result, no effective therapies are available. In our current study, we found that the septic mice presented long-term anxiety and depression induced by CLP. Sickness behaviors, a term which refers to physiological and behavioral changes induced by infection, can also be detected in SAE patients and manifest as depression-related symptoms such as anxiety, anorexia and anhedonia (Dantzer, 2004; Pereira De Souza Goldim et al., 2020). However, sickness behaviors mainly occur in the acute phase of sepsis (Dantzer, 2004). Depression-related symptoms after sepsis are also detected in septic survivors 2–3 months after recovery from the sickness state (Mostel et al., 2019). Therefore, we examined the behavior of septic mice 14 days after CLP, when there was no difference in TD between the sham and CLP group. Furthermore, increased apoptosis and necroptosis in the hippocampus were detected in these mice, resulting in substantial neuronal loss. We demonstrated that apoptosis and necroptosis were related according to Ramsay’s rule and that inhibiting only apoptosis or necroptosis failed to rescue the cognitive symptoms in the septic mice. Importantly, we confirmed that MD2 regulates both apoptosis and necroptosis, and a peptide targeting MD2 reduced both apoptosis and necroptosis while significantly relieving anxiety and depression-like behaviors in the septic mice.

Major depressive disorder (MDD) is common, affecting approximately 16% of the population worldwide, and is associated with a high disability rate and socioeconomic consequences (Zanos et al., 2016). The incidence of long-term depression in septic patients is even higher than that in the general population, but little is known about the underlying mechanism. Evidence from imaging, postmortem and other studies suggests that abnormal corticolimbic structures, including the prefrontal cortex, anterior cingulate cortex, amygdala, and hippocampus, are responsible for MDD (Drevets et al., 2008). Significant neuronal loss in the hippocampus of individuals with MDD has been confirmed (Lee et al., 2002). Moreover, the expression of apoptosis-associated genes was increased in the frontal cortex of MDD patients. This evidence indicates a close link between corticolimbic neuronal death and depression. However, it is still unclear whether neuronal death is the cause or result of depression. Notably, neuronal loss has already been documented in patients with sepsis. Changes in brain volume, especially atrophy of the hippocampus and cortex, have been reported in sepsis with abnormal neurological symptoms (Stubbs et al., 2013). Some studies have reported that inhibition of the cell death pathway can alleviate long-term cognitive impairments and depression-like behaviors in septic mice (Fu et al., 2019; Xu X. E. et al., 2019). In the current study, we found a substantial increase in apoptosis- and necroptosis-associated proteins in the first 3 days after CLP. Accordingly, we also found notable neuronal death in the hippocampus of the mice with sepsis. On the fourteenth day after CLP, the fundamental state was similar to that of the sham-treated mice, indicating that sepsis had been alleviated. However, most of the septic mice showed severe depression-like behaviors. This finding indicated that irreversible severe neuronal death in the hippocampus caused by sepsis may be a main cause of the depression-like behaviors.

Multiple PCDs contribute to the loss of hippocampal neurons. Apoptosis and necroptosis are the main types of PCD that are involved in the pathology of anxiety and depression (Zhang et al., 2020). Multiple studies have confirmed the increased apoptosis in the hippocampus of mice with SAE (Zhou et al., 2020), which is consistent with our results. And many drugs target on the decrease of apoptosis show significant brain-protective effects (Bedirli et al., 2018; Zhou et al., 2020). However, our study showed no benefits of solely inhibiting apoptosis with Z-VAD-FMK or necroptosis with Nec-1. In fact, these drugs not only act on apoptosis but also mediate other mechanisms, such as autophagy, oxidative stress and neuroinflammation, and thus do not directly prove the protective effects of apoptotic inhibition. No studies have been conducted on necroptosis in SAE. However, some studies have focused on the role of necroptosis in the peripheral organs of septic mice. After Nec-1 treatment, the serum levels of proinflammatory factors such as IL-6, IL-1β, and TNF-α were decreased (Bolognese et al., 2018). However, some studies have shown that Nec-1 accelerates death in a rat model of CLP and substantially increases the expression of cleaved caspase-3 in hepatocytes (Zhang et al., 2019). Therefore, the role of necroptosis in sepsis and SAE remains unclear. Currently, increasing evidence has shown the mutual transformation between apoptosis and the necroptosis signaling pathway. Our data showed that both apoptosis and necroptosis participated in the pathological mechanisms of SAE. Interestingly, apoptosis and necroptosis are related through Ramsay’s rule. This phenomenon indicates that inhibition of just one pathway cannot effectively rescue depression. Notably, in the current study, simultaneous treatment with inhibitors of apoptosis and necroptosis caused more death in the septic mice, which may result from the superposition of toxic effects that exceed their protective effects.

Alternatively, we searched for upstream pathways that can simultaneously regulate apoptosis and necroptosis. Our previous study confirmed that MD2, which acts as an accessory protein of TLR4, can also regulate various cell death pathways (Fang et al., 2021). We constructed CaMKII-MD2^fl/fl^ mice with specific knockout of MD2 in excitatory neurons. In the present study, we found significant improvements in the depression-like behaviors of the CaMKII-MD2^fl/fl^ mice with sepsis. However, the MD2-TLR4 complex has been widely explored in sepsis. MD2 was responsible for the recognition and bonding of the membrane component of gram-negative bacteria (lipopolysaccharide) and triggering the TLR4-mediated inflammatory response. Inhibitors of MD2 could reduce inflammation and alleviate tissue injury in sepsis (Chen et al., 2017). As the effects of neuroinflammation on MDD have been potently confirmed (Brites and Fernandes, 2015), we could not ignore the effect of MD2 blockade on inflammation in the hippocampus of mice with sepsis. Some systematic reviews and meta-analyses have shown that only IL-6 expression is significantly higher in depression (Ng et al., 2018). In accordance with these results, our findings demonstrated that HMGB1 and IL-6 levels were significantly decreased in the CaMKII-MD2^fl/fl^ mice compared with the WT mice at 24 h after CLP.

The molecular mechanism by which MD2 regulates apoptosis and necroptosis remains unknown and needs further exploration. Our previous study found that MD2 mediated apoptosis and necroptosis by combining with Src-associated substrate in mitosis of 68 KD (Sam68). TAT-CIRP restrained the function of MD2 and reduced brain injury-induced stroke (Fang et al., 2021). In this study, we used TAT-CIRP at a dose of 25 mg/kg in the brain to reduce neuronal death and effectively relieve SAE symptoms. Sam68, also known as KHDRBS1, participates in the cell cycle, apoptosis and signaling. Sam68 was confirmed to be recruited to the TNF receptor and promote the recruitment and ubiquitylation of RIP (Ramakrishnan and Baltimore, 2011). Moreover, Sam68 is a part of the TNF-induced cytoplasmic caspase-8-FADD complex, and its cleavage by activated caspase, especially caspase-8, triggers the apoptosis pathway (Cho et al., 2015). However, the mechanism by which MD2 acts on Sam68 and whether other molecules can interact with MD2 remain unclear. More studies on the regulatory mechanisms of MD2 will contribute to its use in the clinic for sepsis.

In our study, MD2-perturbing peptide (TC) was systematically administrated to mice after CLP. Treatment with TC increased the survival rate of CLP mice and reduced anxiety-like behaviors, which indicates its benefits for facilitating recovery from sepsis. One possible mechanism of these benefits is the reduction in multiorgan damage, because MD2 is abundantly expressed in peripheral organs such as the heart, kidney and liver and mediates proinflammatory responses (Xu S. et al., 2019; Wang et al., 2020). Further investigation is needed to determine whether inhibition of MD2 reduces tissue damage by inhibiting cellular apoptosis and necroptosis in sepsis. In addition, it remains unclear whether treating SAE with intraperitoneal injections of TC is due to TC itself or reductions in sepsis. In some studies, systemic administration of melatonin improved the survival rate of CLP mice and alleviated sepsis-associated organ dysfunction including brain disorders, which indicates possible beneficial effects of TC on treating sepsis in SAE patients (Zhao et al., 2015; Ji et al., 2018).

There are some limitations in this study. First, the CaMKII-MD2^fl/fl^ mice had MD2 knocked out in the neurons of the whole brain and TC was administered *via* icv injection. Therefore, we could not eliminate the influence of other structures, such as the cortex and amygdala, which are associated with depression. Further studies exploring MD2-mediated neuronal death in the cortex and amygdala are needed. Next, we confirmed the relationships among neuronal death, neuroinflammation and depression in septic mice. Whether neuroinflammation is the cause or the result of cell death is still unknown and needs further investigation. Finally, MD2 is expressed not only in neurons but also in microglia and astrocytes in the brain. MD2 in peripheral immune cells can also enter the brain through the damaged brain-blood barrier in septic mice. Thus, we could not ignore the effects of extraneous MD2 on neurons. These issues related to MD2 in sepsis need further exploration.

## Conclusion

In summary, the present study demonstrated for the first time that neuronal apoptosis and necroptosis in the hippocampus contribute to depression-like behaviors in septic mice. We demonstrated that MD2 was the crosstalk mediator of apoptosis, necroptosis, and neuroinflammation in the pathology of depression induced by sepsis. Inhibition of MD2 in neurons can reduce neuronal loss and alleviate depression-like behaviors in septic mice. Methods targeting MD2 in neurons may provide new treatment strategies for SAE.

## Data Availability

The original contributions presented in the study are included in the article/Supplementary Material, further inquiries can be directed to the corresponding authors.

## Abbreviations

CLP, cecal ligation and puncture; CNS, central nervous system; DAMP, damage-associated molecular pattern; EPM, elevated plus maze; FST, forced swimming test; MDD, major depressive disorder; MD2, Myeloid differentiation factor 2; MSS, murine sepsis score; OFT, open field test; PCD, programmed cell death; PVC, polyvinyl chloride; SAE, sepsis-associated encephalopathy; Sam68, Src-associated substrate in mitosis of 68 KD; TC, TAT-CIRP; TD, total distance.
