# Supplementary material for: Neuronal MD2 induces long-term mental impairments in septic mice by facilitating necroptosis and apoptosis
Source: Front Pharmacol. 2022 Aug 9;13:884821. doi: 10.3389/fphar.2022.884821 (PMC9396348; doi:10.3389/fphar.2022.884821)
Supplement: Supplementary file 1 [file Datasheet2.PDF]

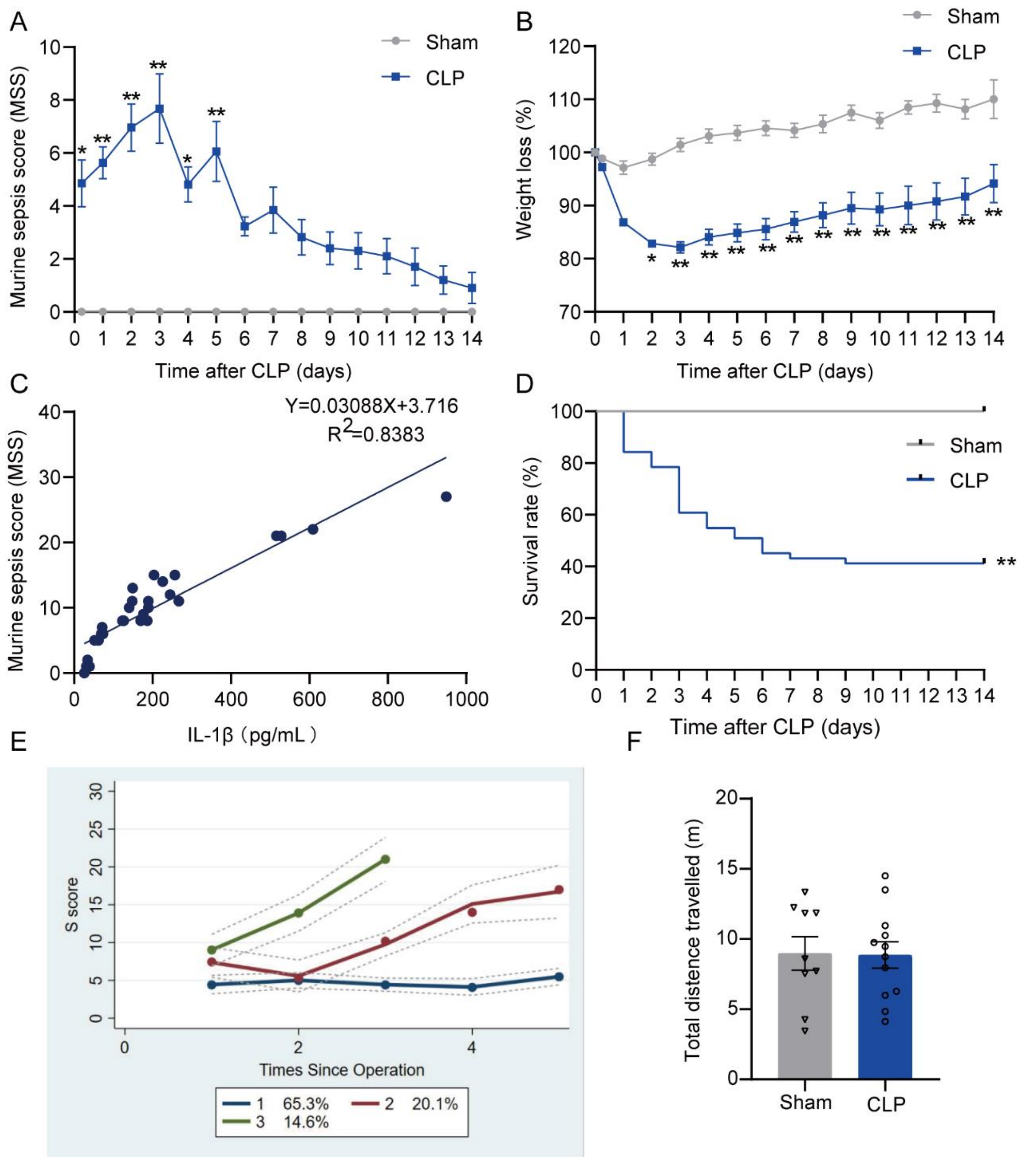

Supplementary Fig.1. The generation and evaluation of SAE mouse models

A. The MSS of different groups over time (n CLP=24). **P*<0.05, ***P* < 0.01 *vs.* the sham. Data are shown as the mean ± SEM. B. Changes in weight among different groups (n Sham=13, n CLP=28). **P*<0.05, ***P* < 0.01 *vs*. the sham. Data are shown as the mean ± SEM. C. Correlation analysis between MSS and serum IL-1β level (n=25). D. Survival curve for CLP-induced mice (n=59). E. The group trajectory development model according to the MSS and final outcome. F. The total distance traveled of the sham and CLP groups (n sham=9, n CLP=12). Data are shown as the mean ± SEM.

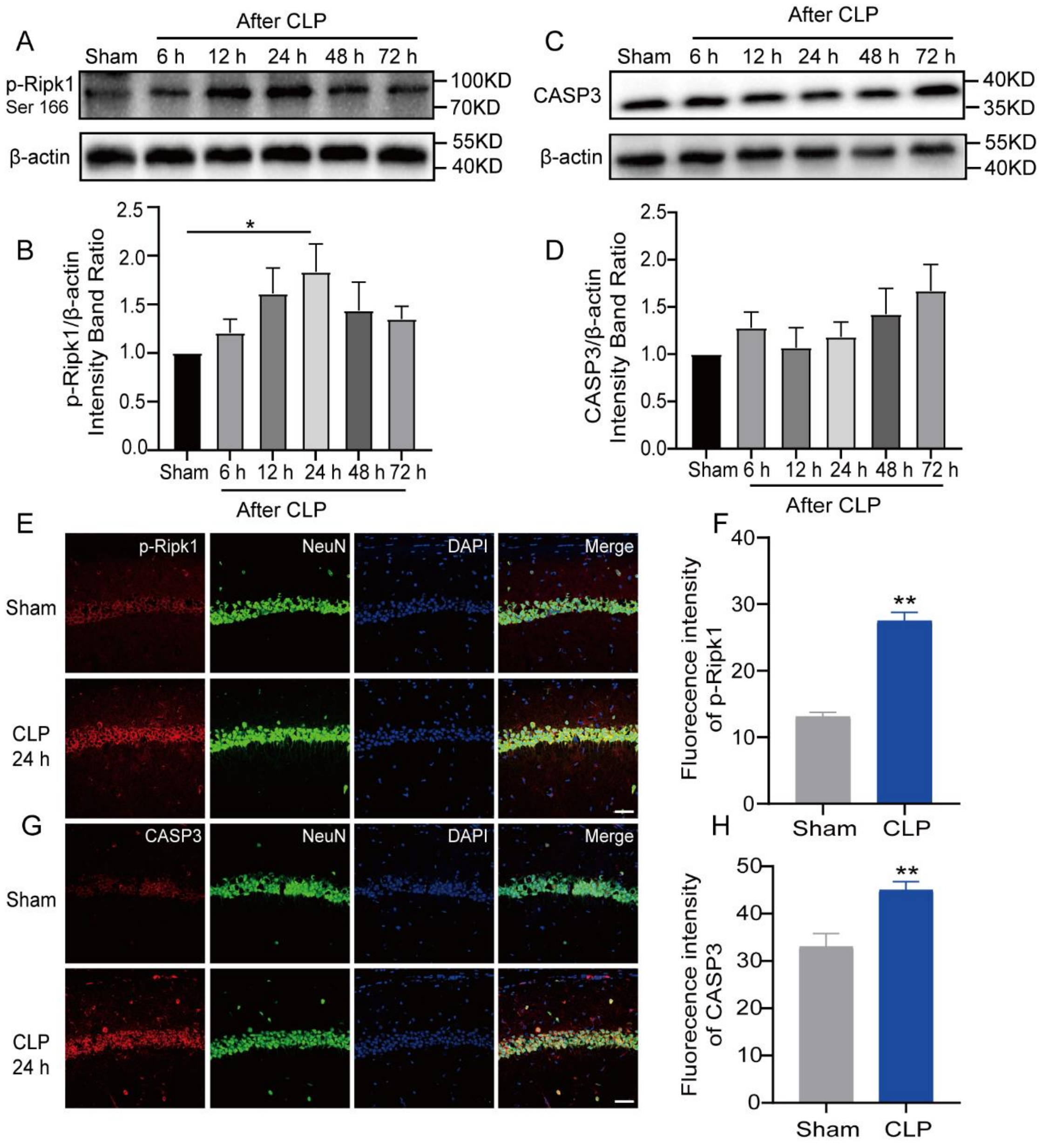

Supplementary Fig. 2. Apoptosis and necroptosis were increased in the hippocampus of mice with sepsis

A. Representative Western blot of p-Ripk1. B. Quantitative evaluation of p-Ripk1 expression after CLP at the time point. *P<0.05 *vs.* the sham. Data are shown as the mean ± SEM (n=9). C. Representative Western blot of caspase-3. D. Quantitative evaluation of caspase-3 expression after CLP at the time point. Data are shown as the mean ± SEM (n=9). E. IF staining of p-Ripk1 24 h after CLP (bar=40 μm). F. Analysis of the intensity of p-Ripk1 between the sham and CLP groups. **P<0.01 *vs.* the sham. Data are shown as the mean ± SEM (n=9). G. IF staining of caspase-3 24 h after CLP (bar=40 μm). H. Analysis of the intensity of caspase-3 between the sham and CLP. **P<0.01 *vs.* the sham. Data are shown as the mean ± SEM (n=9).

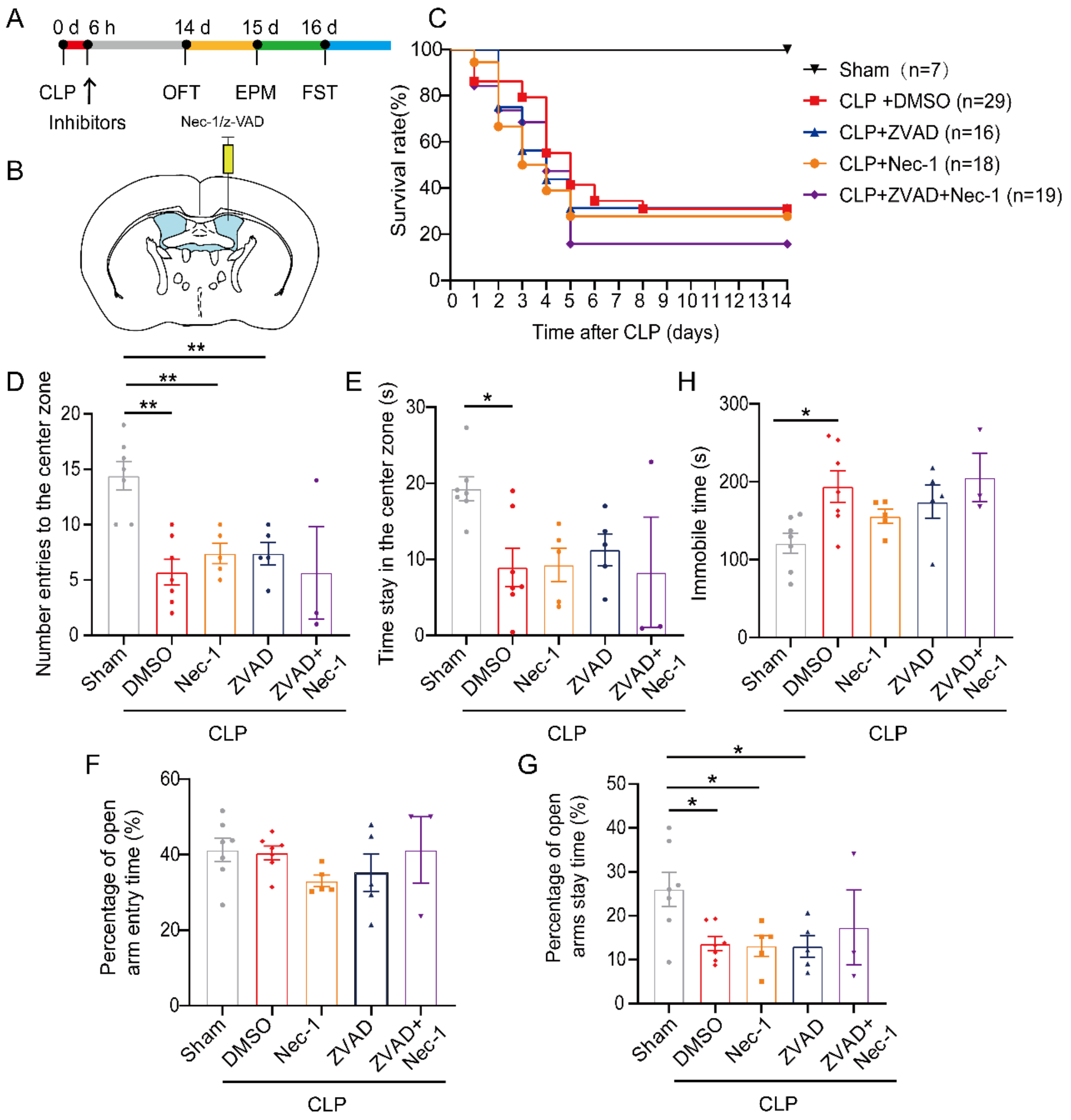

Supplementary Fig. 3. Inhibition of apoptosis or necroptosis resulted in no difference in depression-associated behaviors

A. An outline of the experimental procedure for inhibitor injection and behavioral tests of mice with sepsis. B. Schematic configuration of icv injection. C. The survival rate of different cell death inhibitors. D. The effects of different treatments on the number of entries to the center zone. E. The time spent in the center zone in the OFT (n sham=7, n CLP+DMSO=7, n CLP+nec-1=5, n CLP+ZVAD=5, n CLP+Nec-1+ZVAD=3). **P*<0.05, ***P* < 0.01. Data are shown as the mean ± SEM. F. In the EPM, the percentage of entries to the open arms. G. The percentage of time spent in the open arms among different groups. **P*<0.05. Data are shown as the mean ± SEM (n sham=7, n CLP+DMSO=7, nCLP+nec-1=5, n CLP+ZVAD=5, n CLP+Nec-1+ZVAD=3). H. Total immobility

time over 5 min in the FST among different groups (n sham= 7, n CLP+DMSO=7, n CLP+nec-1=5, n CLP+ZVAD=5, n CLP+Nec-1+ZVAD=3). *P<0.05. Data are shown as the mean ± SEM.

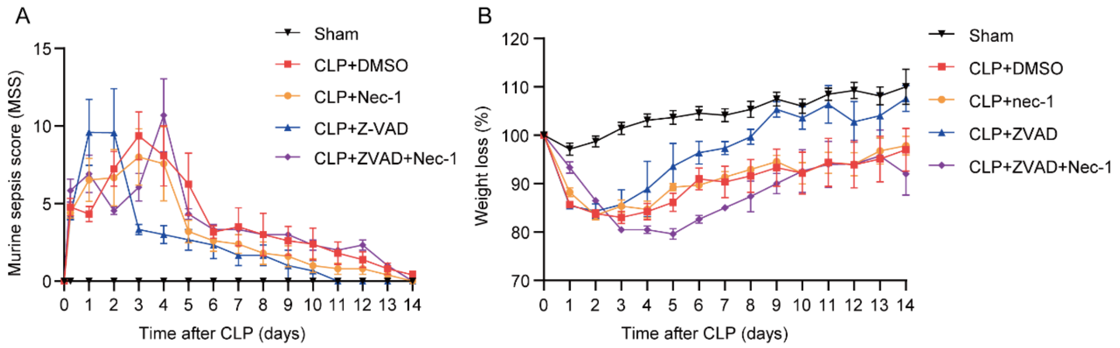

Supplementary Fig. 4. MSS and weight loss of septic mice treated with apoptosis or necroptosis inhibitors

A. The MSS of different groups over time. B. Changes in weight among different groups.

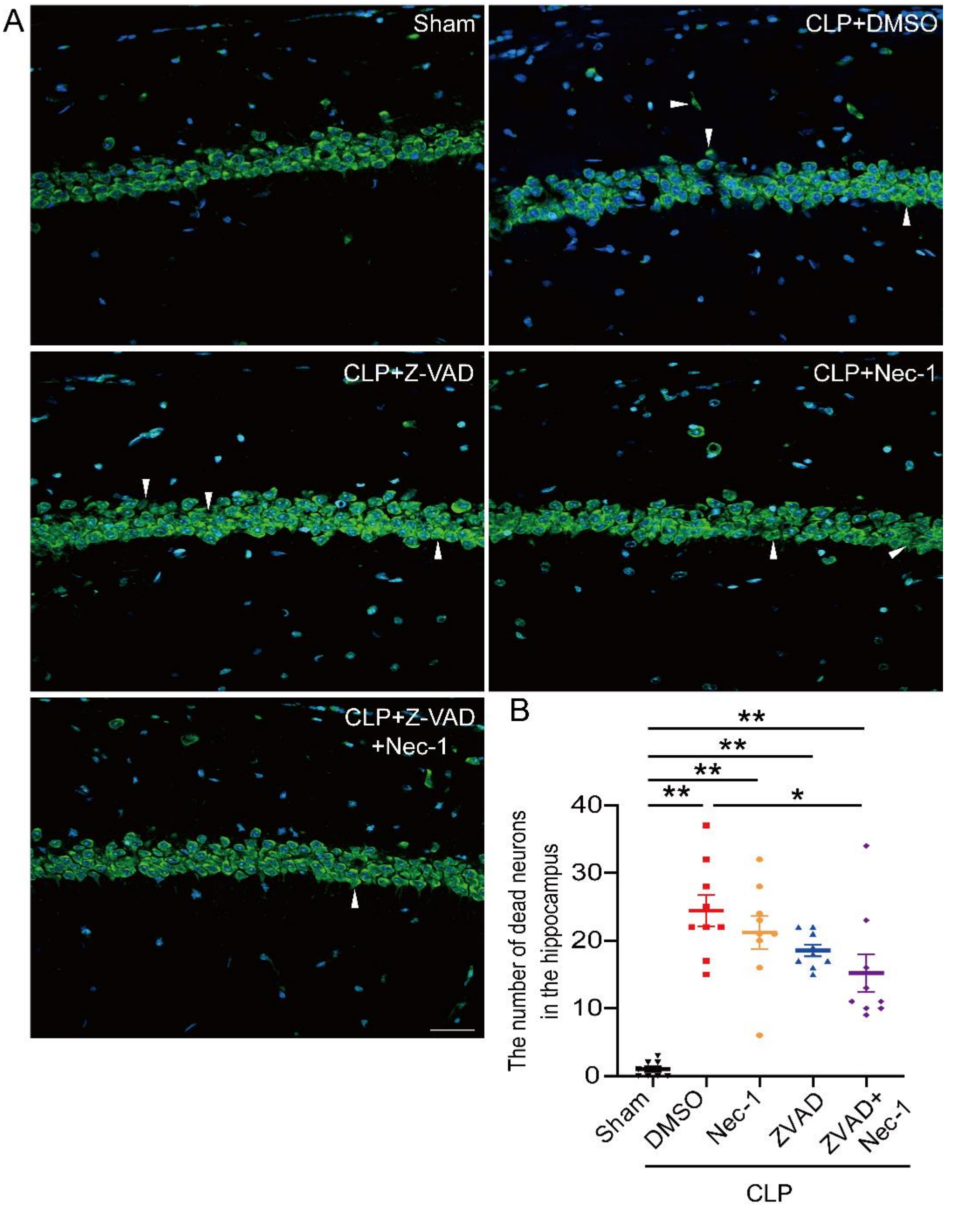

Supplementary Fig. 5. Neuronal death in the hippocampus of septic mice treated with apoptosis or necroptosis inhibitors

A. NeuroTrace^{TM} Nissl staining in the hippocampus at 14 days after CLP. B. The number of dead neurons in the hippocampus among different groups. **P*<0.05, ***P*<0.01. Data are shown as the mean ± SEM (n=9).

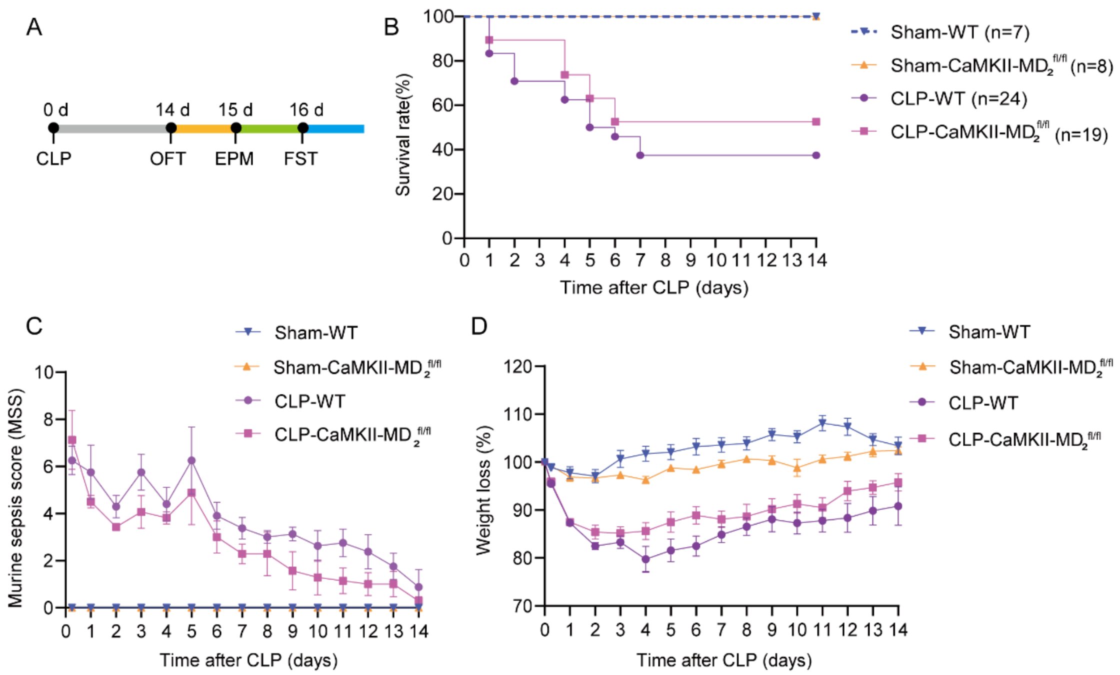

Supplementary Fig. 6. Survival rate, MSS and weight trend of CaMKII-MD2$^{fl/fl}$ mice with sepsis

A. An outline of the experimental procedure for mice with CLP surgery and behavioral tests. B. Survival curve. 9 of 24 mice in the CLP-WT group and 10 of 19 mice in the CLP-CAMKII-MD2$^{fl/fl}$ group survived by day 14. C. The MSS of different groups over time. D. Changes in weight among different groups.

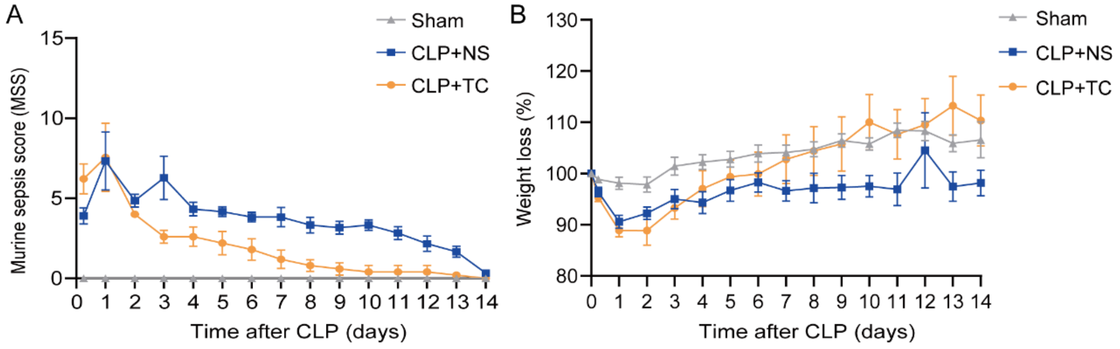

Supplementary Fig. 7. MSS and weight trends of septic mice with icv injection of TC

A. The MSS of different groups over time. B. Changes in weight among different groups.

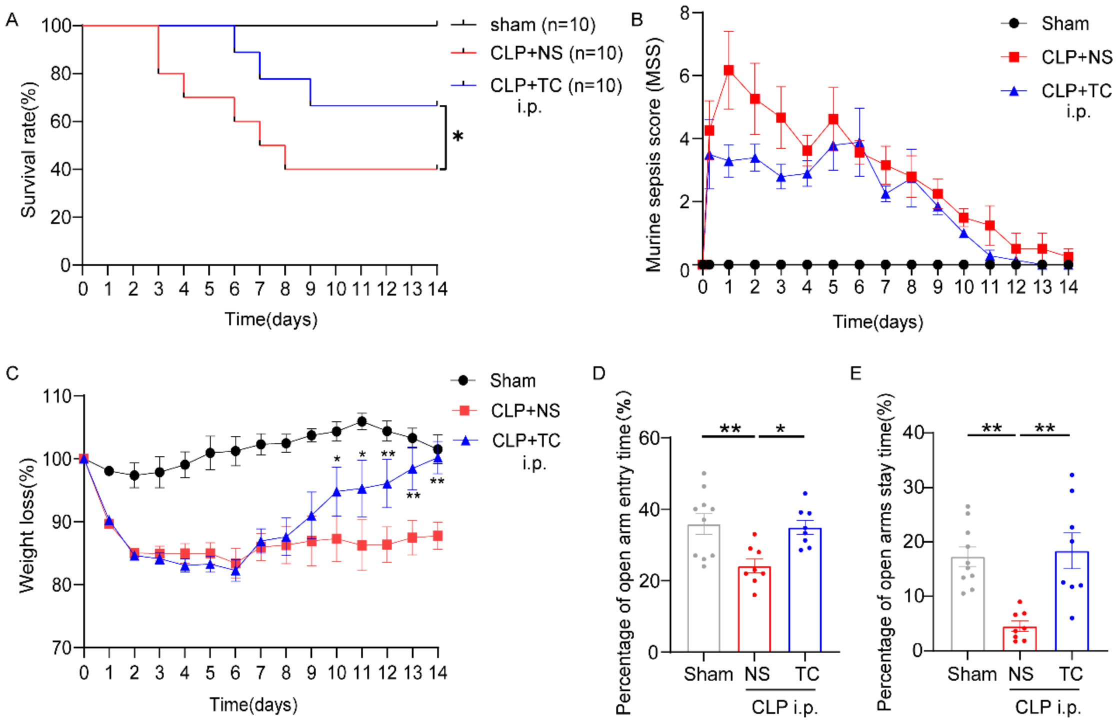

Supplementary Fig. 8. The effects of systemically administration of TC systemically administration on CLP mice

A. Survival curve: 4 of 10 mice in the CLP+NS group and 6 of 10 mice in the CLP+TC group survived by day 14. CLP+NS *vs*. CLP+TC, $*P < 0.05$. B. The MSS of different groups over time. C. Changes in weight among different groups. CLP+NS *vs.* CLP+TC, $*P < 0.05$, $**P < 0.01$. D. EPM result: the percentage of entries into open arms, and G. the percentage of time spent in the open arms among different groups. (n sham=10, n CLP =8, n CLP+TC=8). $*P<0.05$, $**P < 0.01$. Data are shown as the mean ± SEM.
