# Supplementary material for: Neuronal MD2 induces long-term mental impairments in septic mice by facilitating necroptosis and apoptosis
Source: Front Pharmacol. 2022 Aug 9;13:884821. doi: 10.3389/fphar.2022.884821 (PMC9396348; doi:10.3389/fphar.2022.884821)

Figure 1G

Sham

CLP

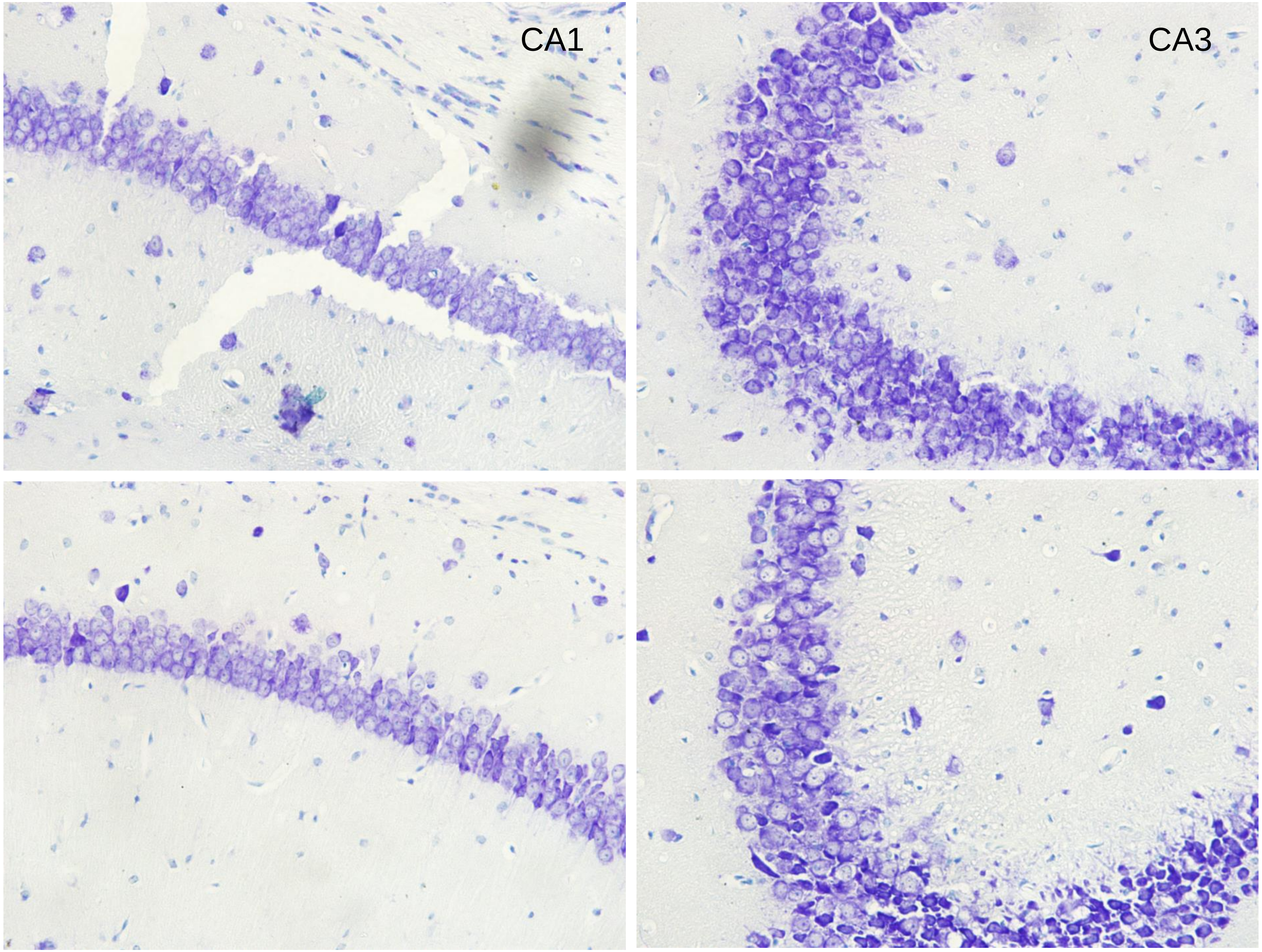

Figure 2E

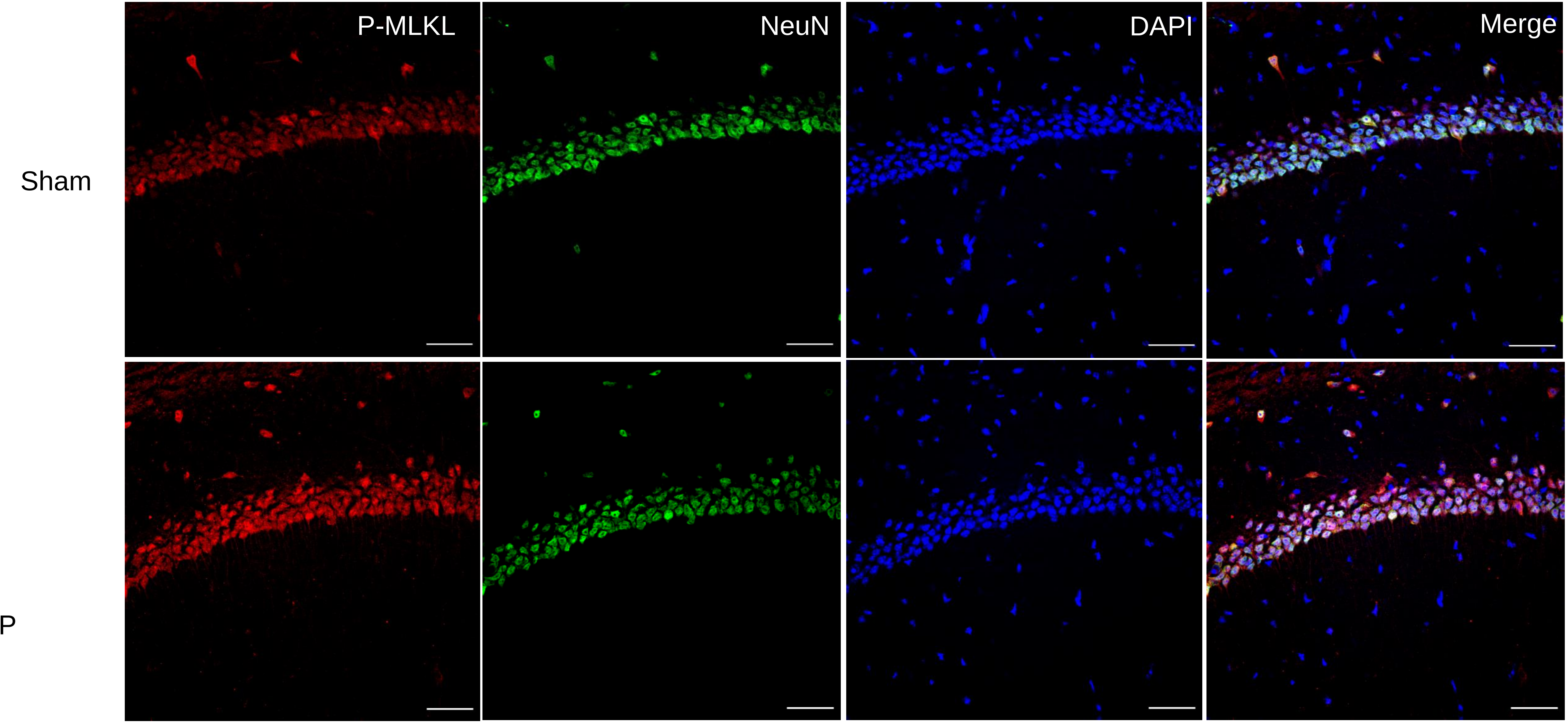

Figure 2G

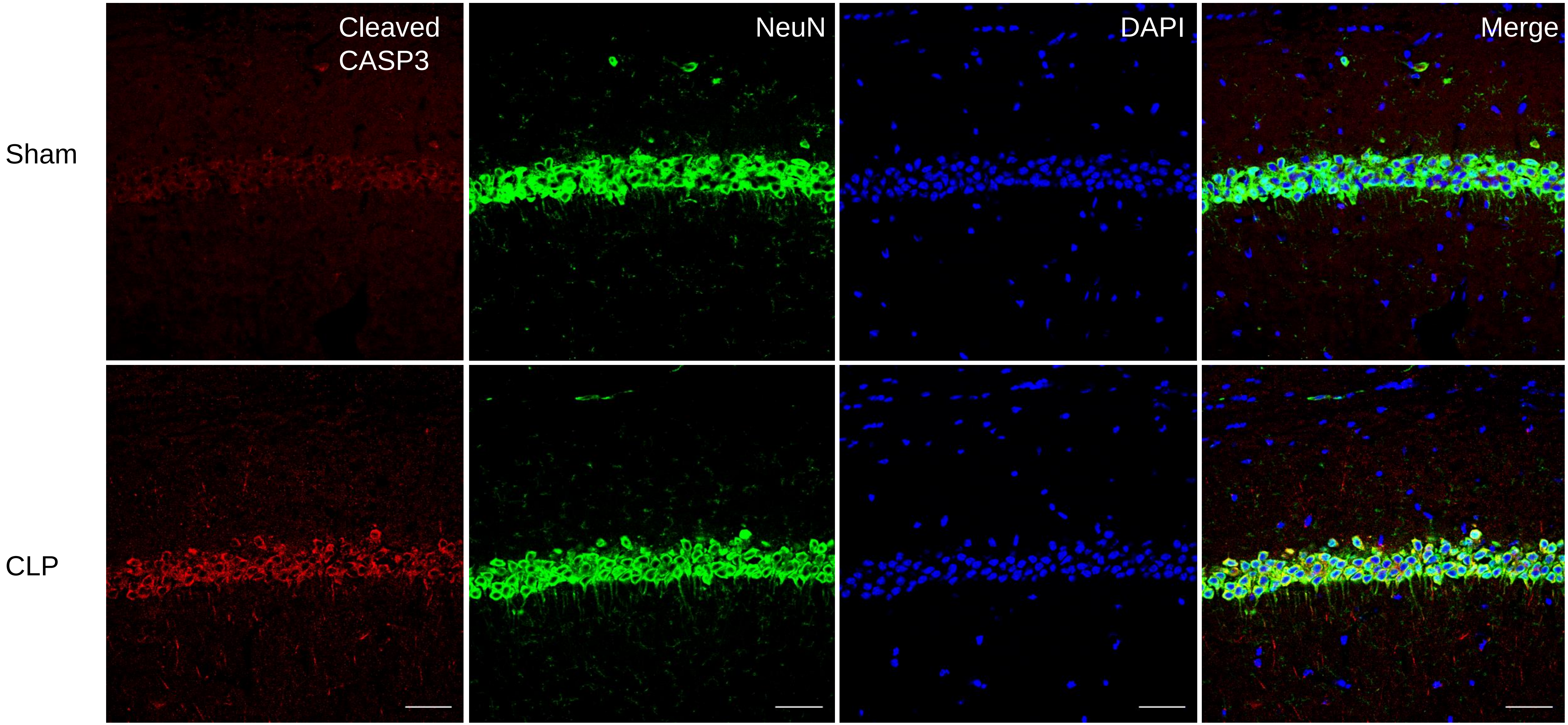

Figure 4C

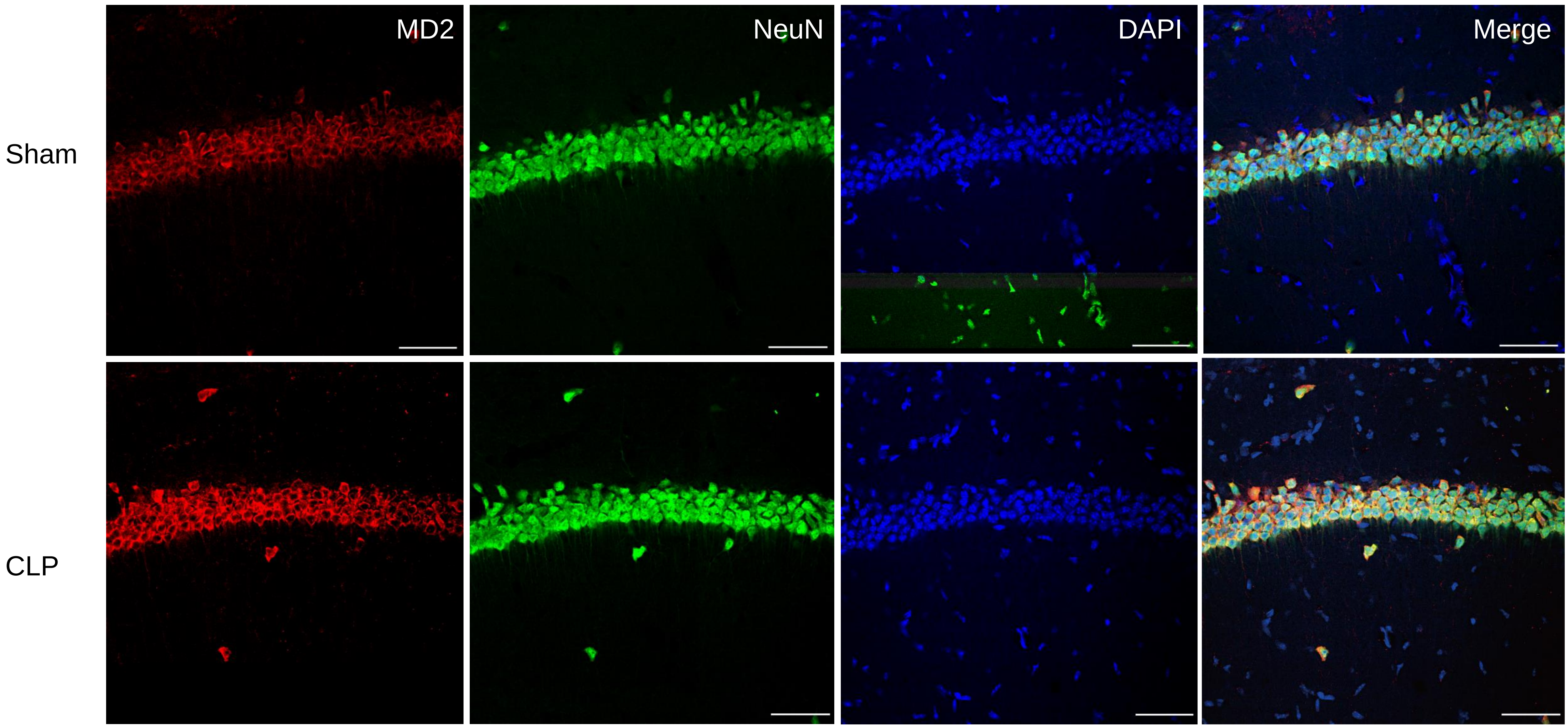

Figure 5G

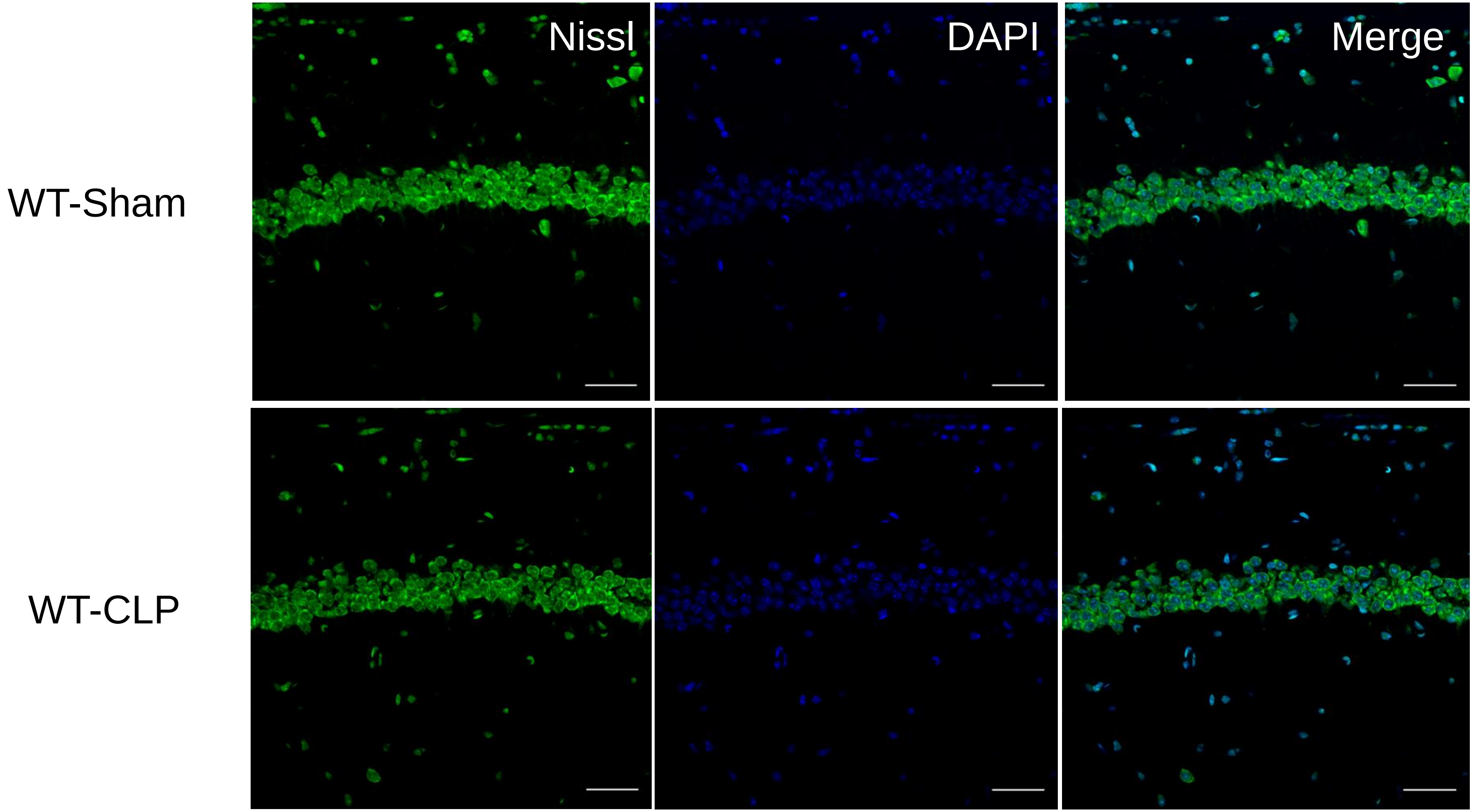

Another two group images were put in next PPT

Figure 5G

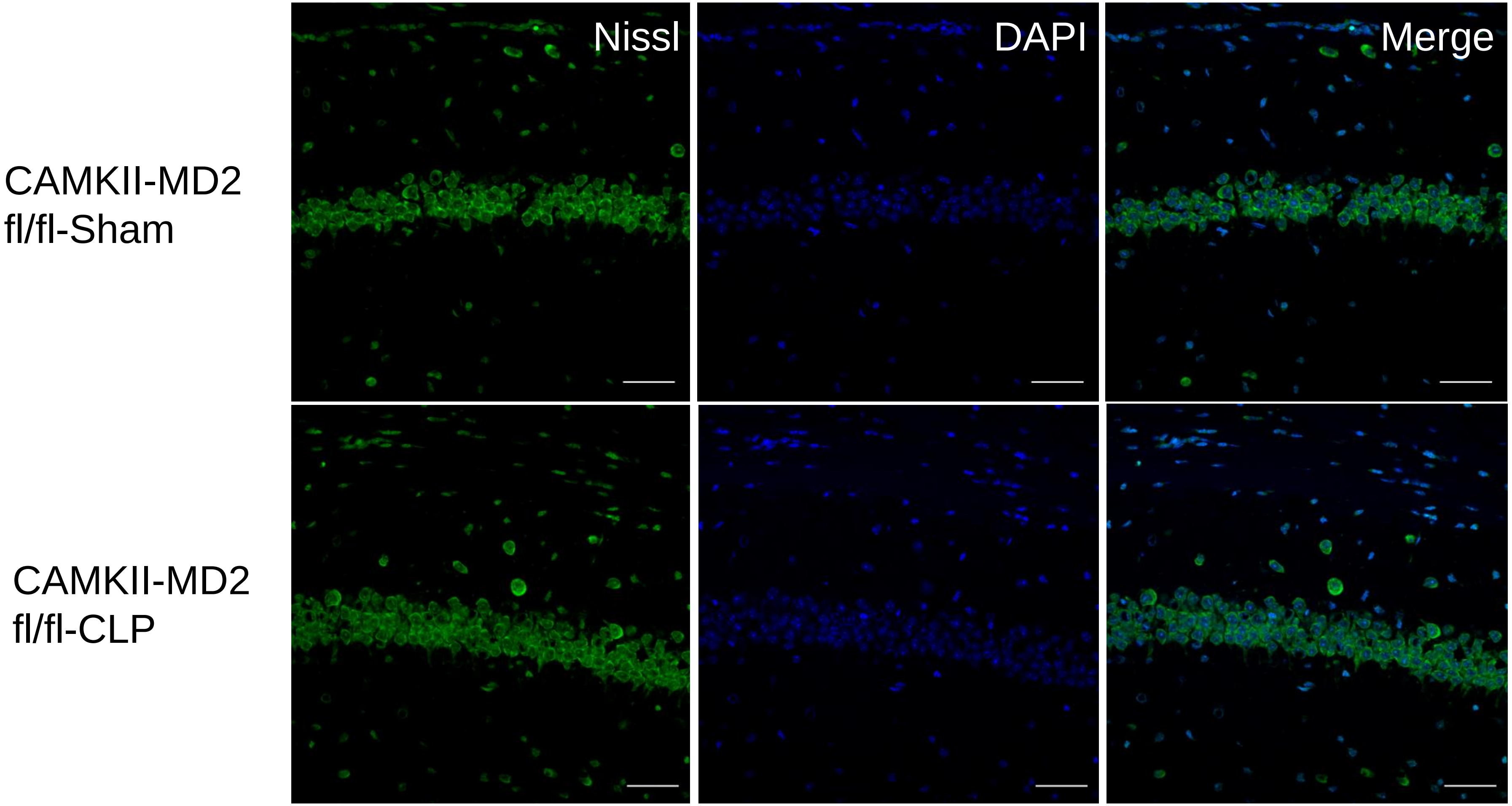

Figure 8A

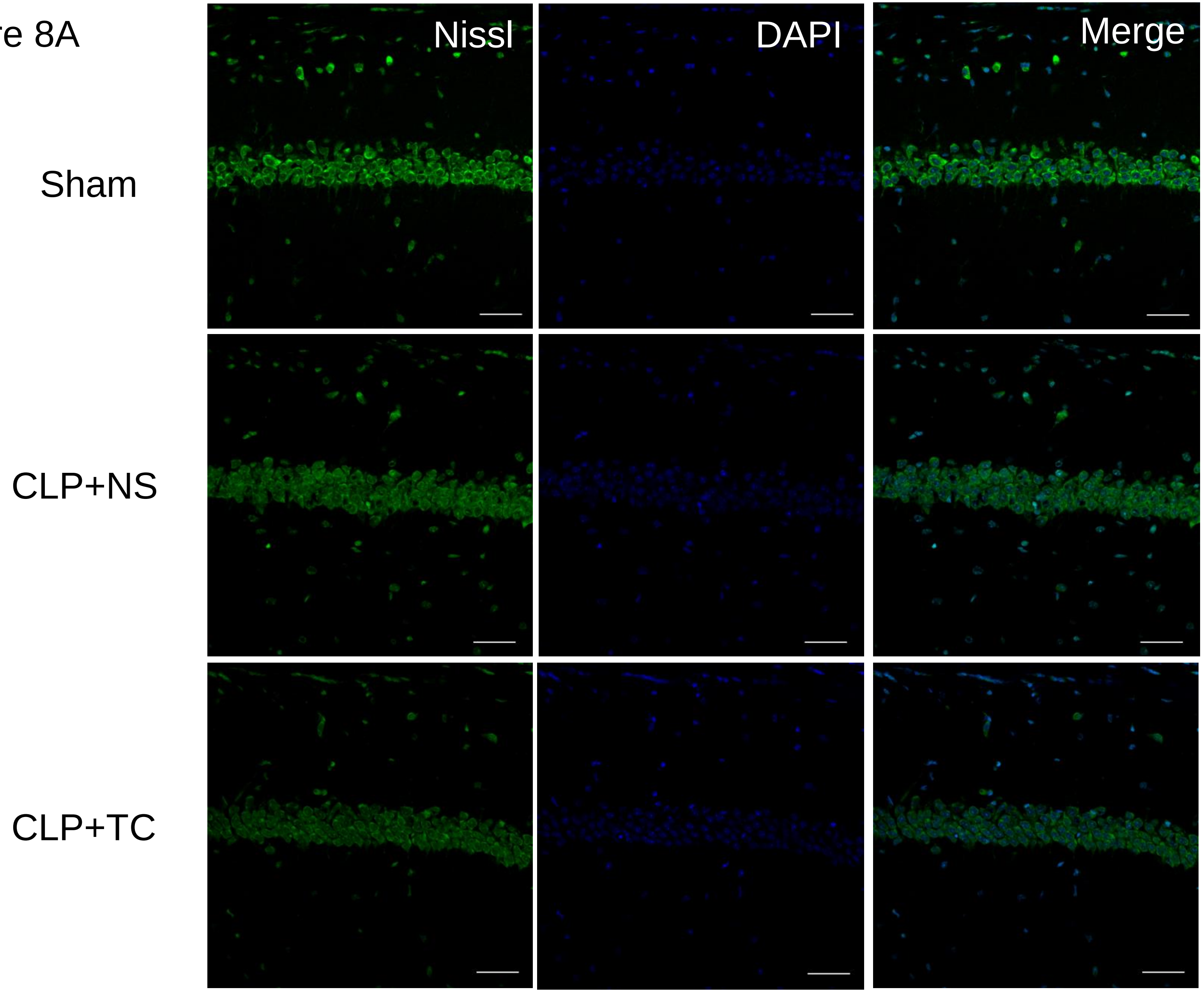

Supplementary Figure 2E

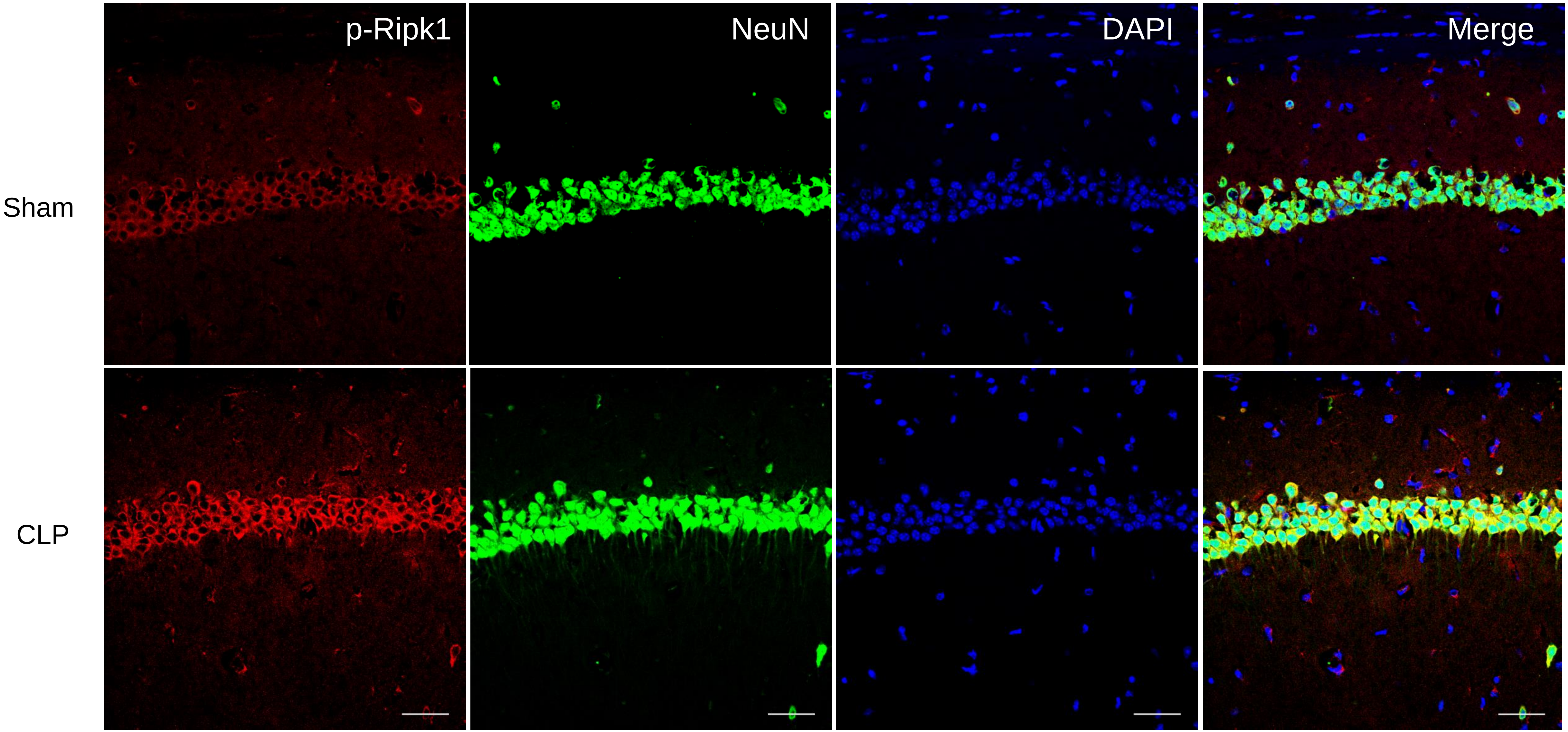

Supplementary Figure 2G

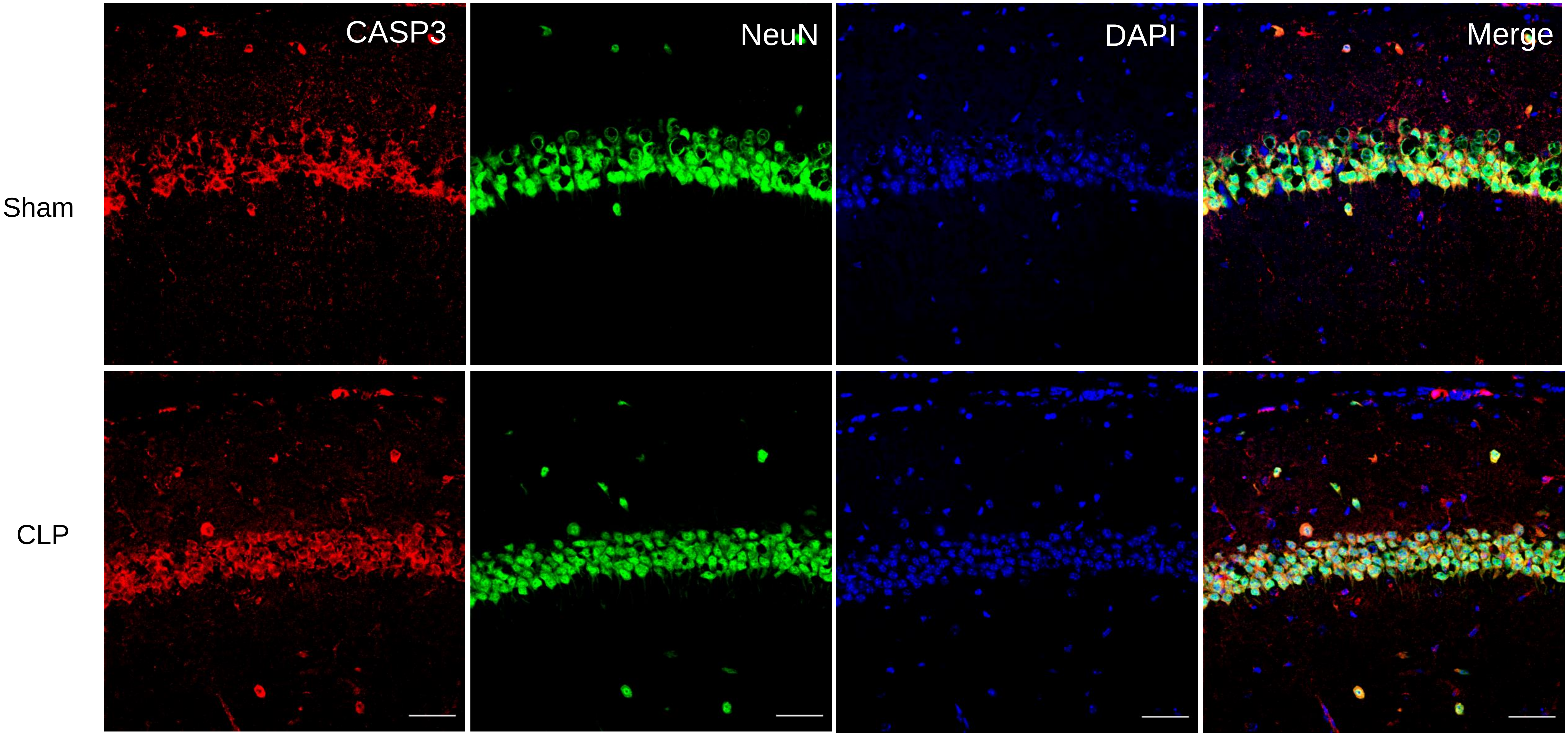

Supplementary Figure 5A

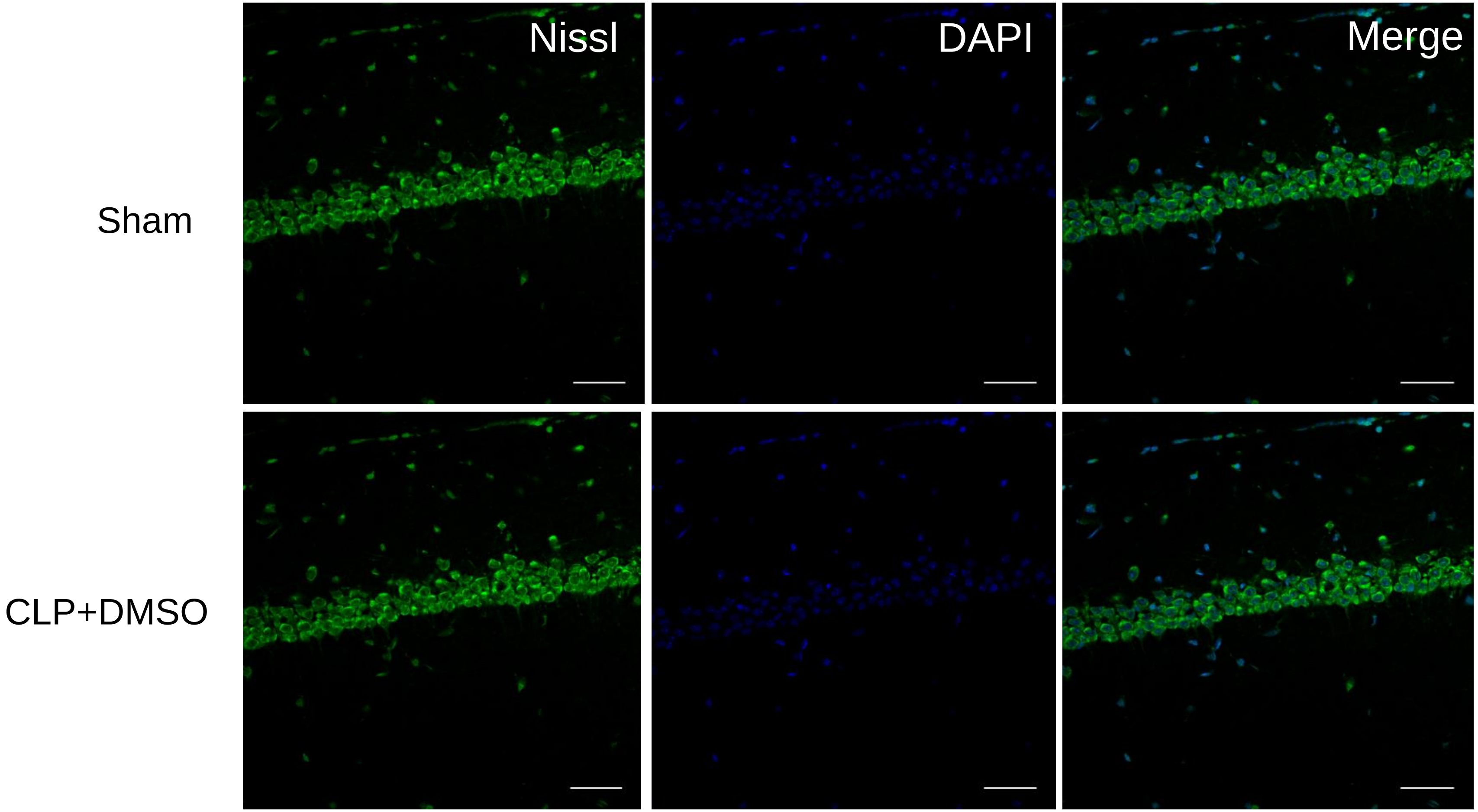

Another three group images were put in next PPT

Supplementary Figure 5A

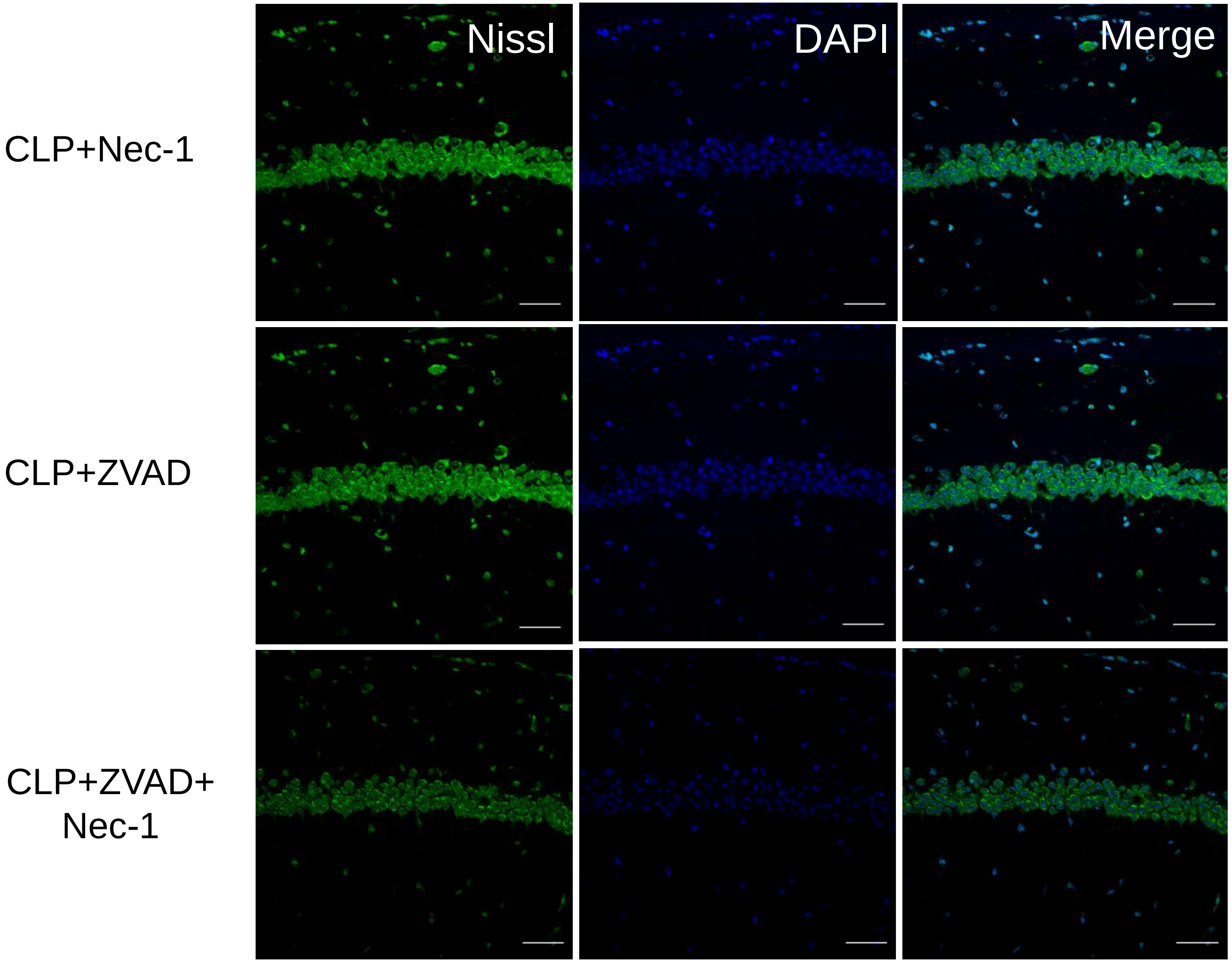

Figure 2A

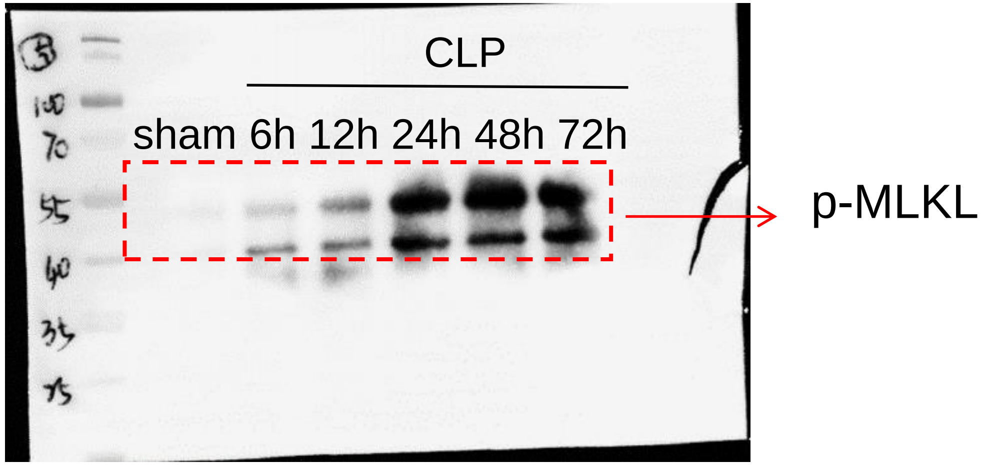
CLP
sham 6h 12h 24h 48h 72h
p-MLKL

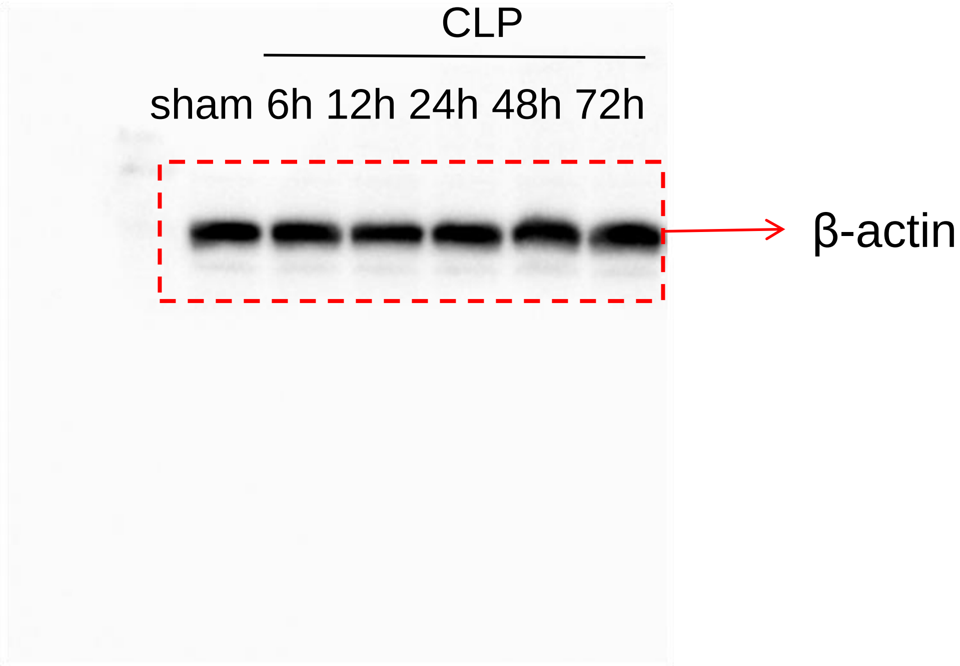
CLP
sham 6h 12h 24h 48h 72h
β-actin

Figure 2B

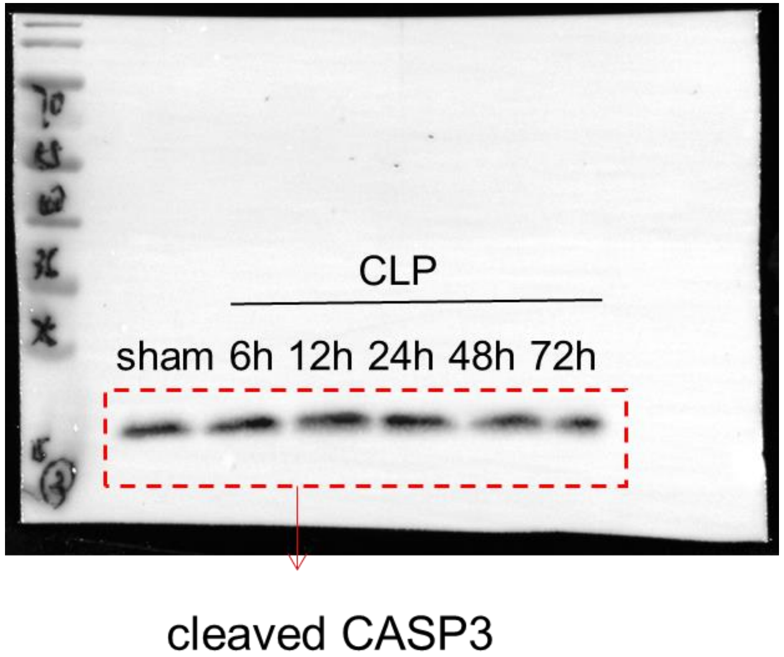
CLP
sham 6h 12h 24h 48h 72h
cleaved CASP3

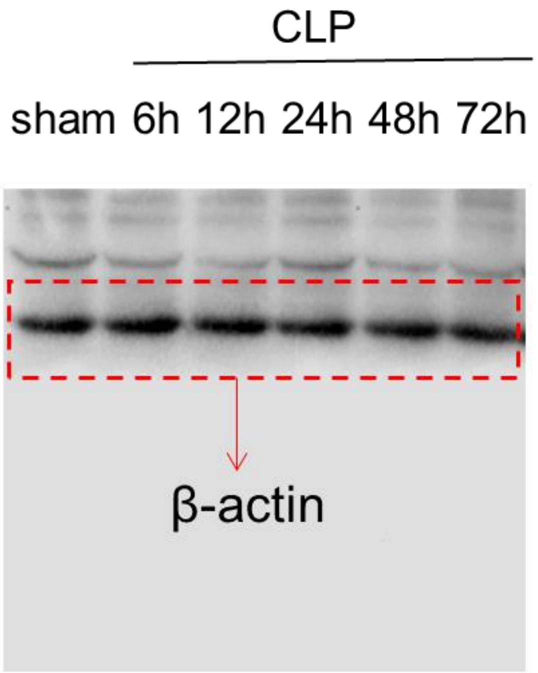
CLP
sham 6h 12h 24h 48h 72h
β-actin

Figure 3A

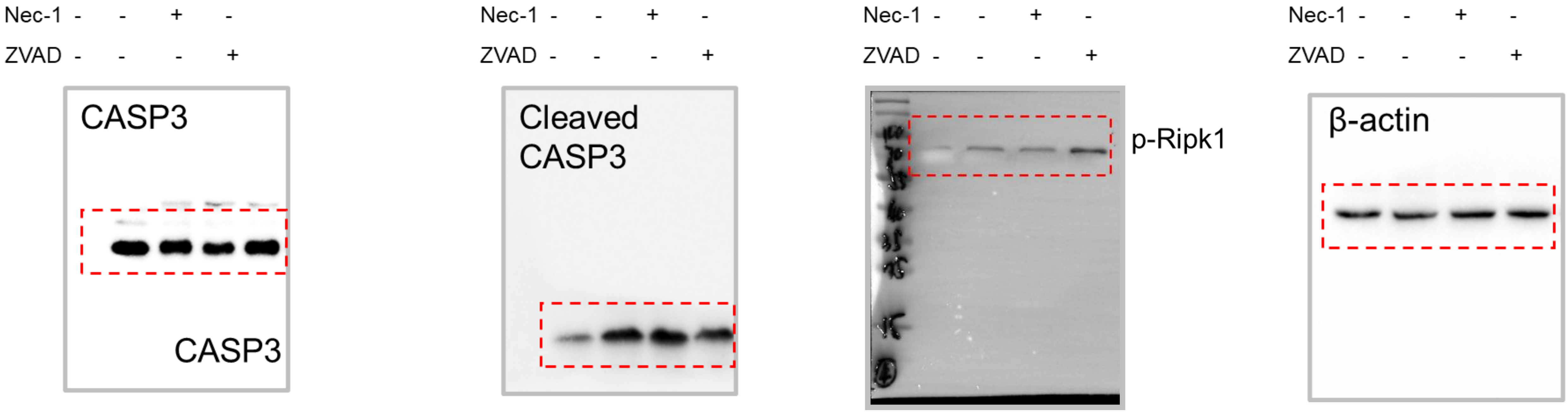
Nec-1 - - + -
ZVAD - - - +
CASP3
CASP3
Nec-1 - - + -
ZVAD - - - +
Cleaved CASP3
Nec-1 - - + -
ZVAD - - - +
p-Ripk1
Nec-1 - - + -
ZVAD - - - +
β-actin

Figure 4A

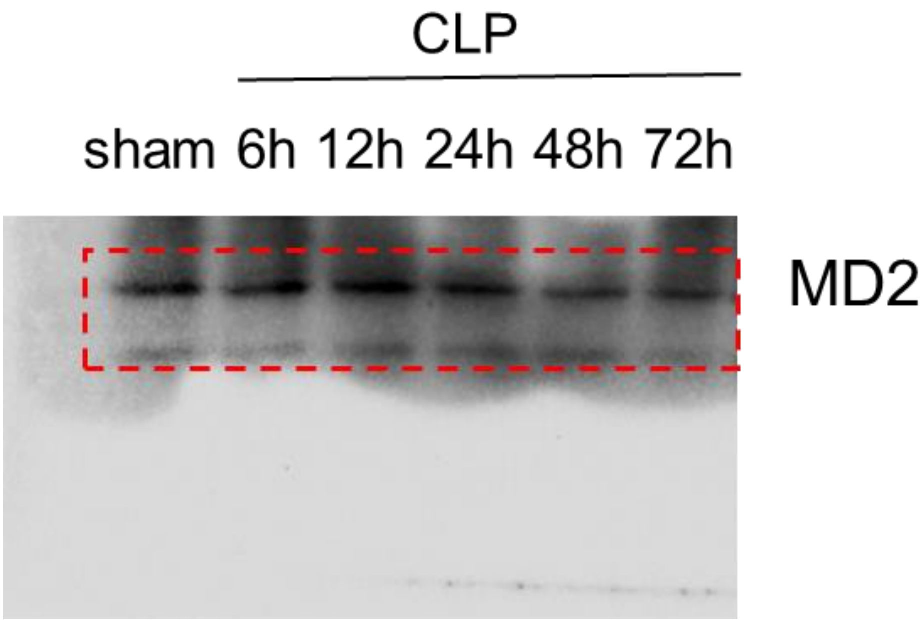
CLP
sham 6h 12h 24h 48h 72h
MD2

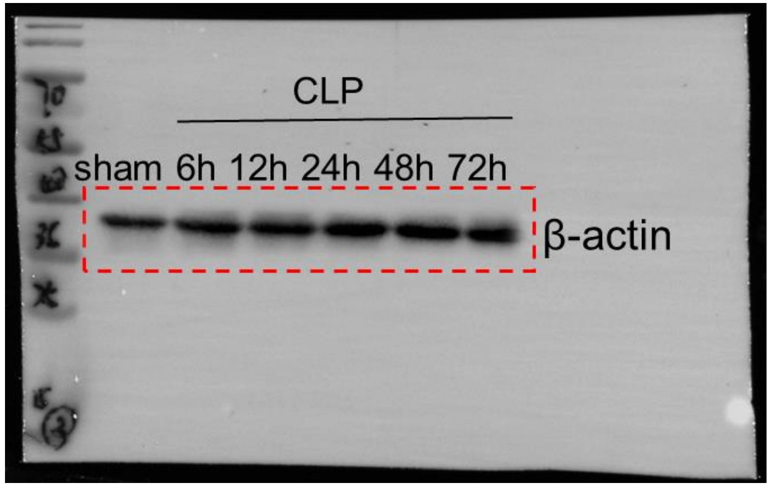
CLP
sham 6h 12h 24h 48h 72h
β-actin

Figure 5A

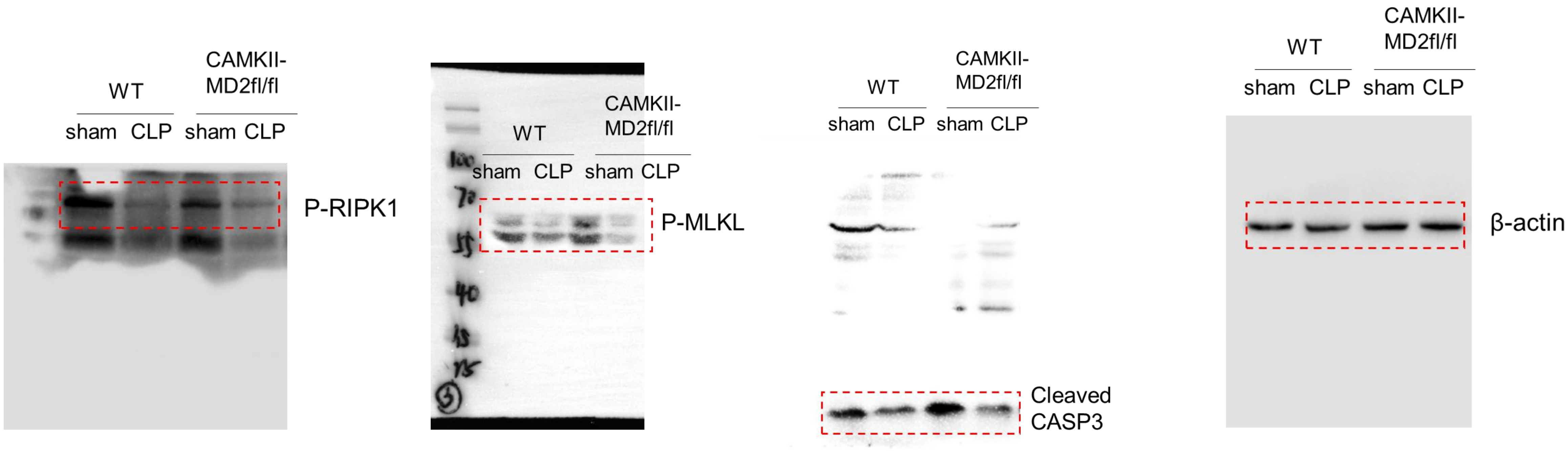
WT
CAMKII-MD2fl/fl
sham CLP sham CLP
P-RIPK1
WT
CAMKII-MD2fl/fl
sham CLP sham CLP
P-MLKL
WT
CAMKII-MD2fl/fl
sham CLP sham CLP
Cleaved CASP3
WT
CAMKII-MD2fl/fl
sham CLP sham CLP
β-actin

Figure 5E

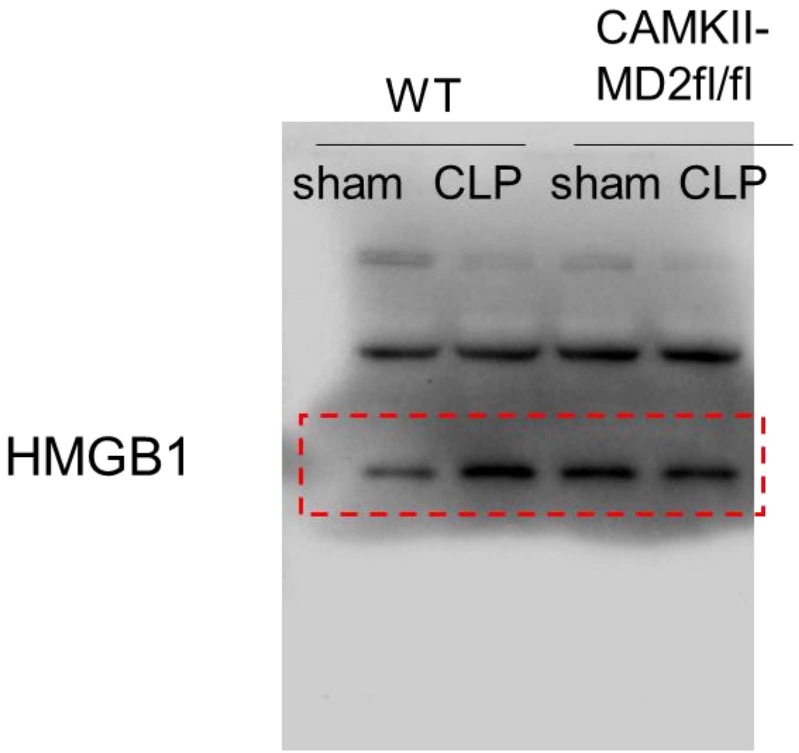
WT
CAMKII-
MD2fl/fl
sham CLP sham CLP
HMGB1

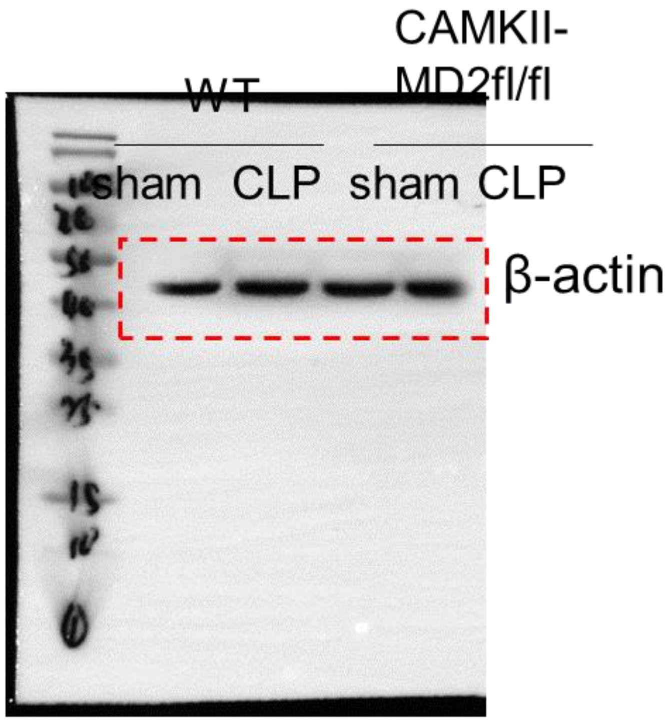
WT
CAMKII-
MD2fl/fl
sham CLP sham CLP
β-actin

Figure 6G

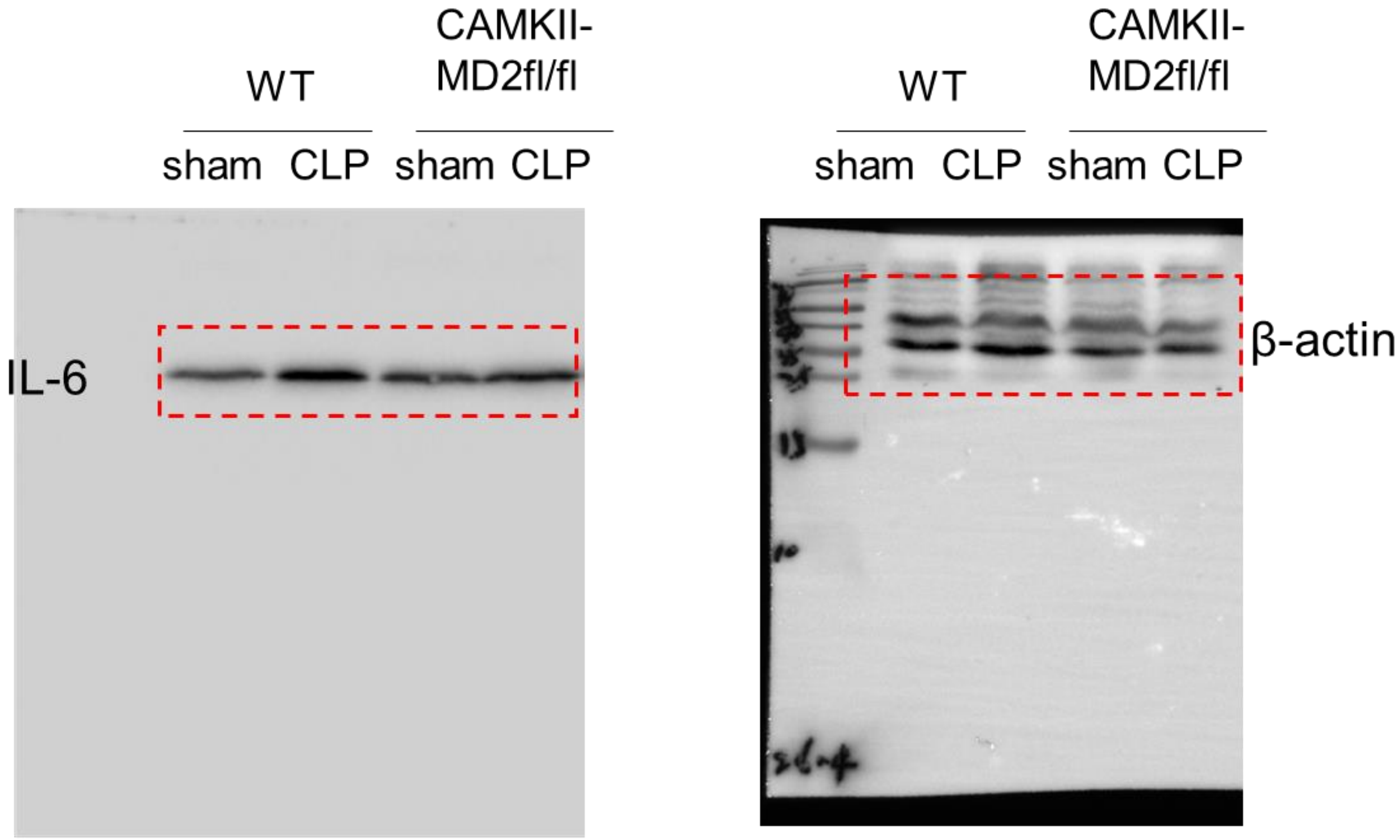
WT
CAMKII-MD2fl/fl
sham
CLP
sham
CLP
IL-6
WT
CAMKII-MD2fl/fl
sham
CLP
sham
CLP
β-actin

Supplementary Figure 2A

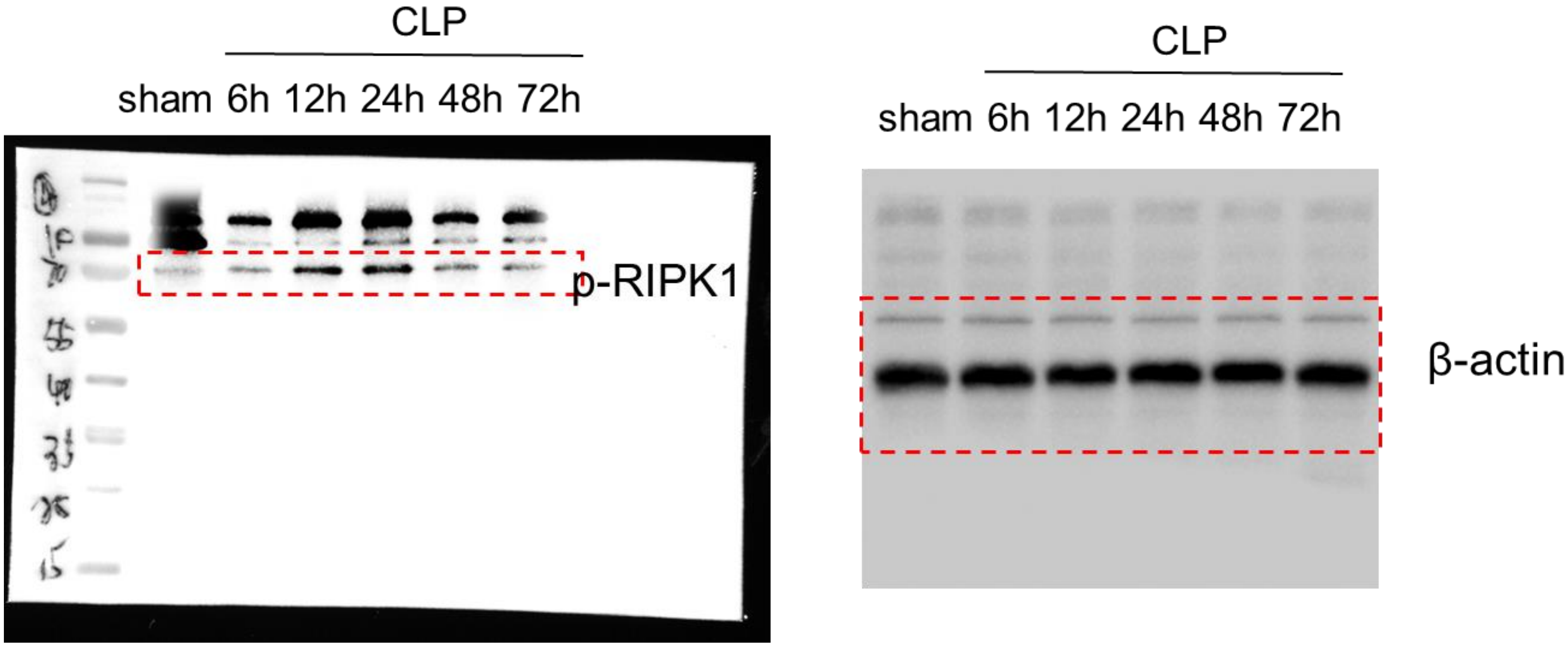

Supplementary Figure 2C

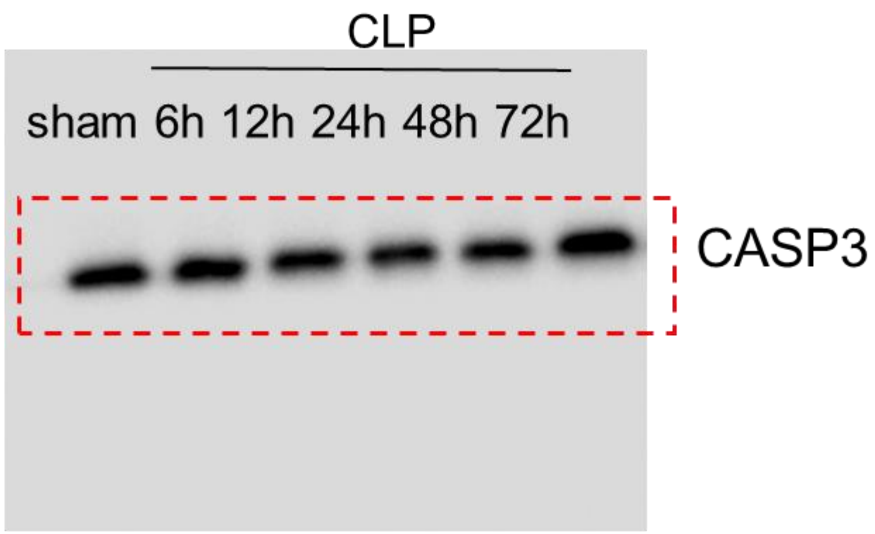

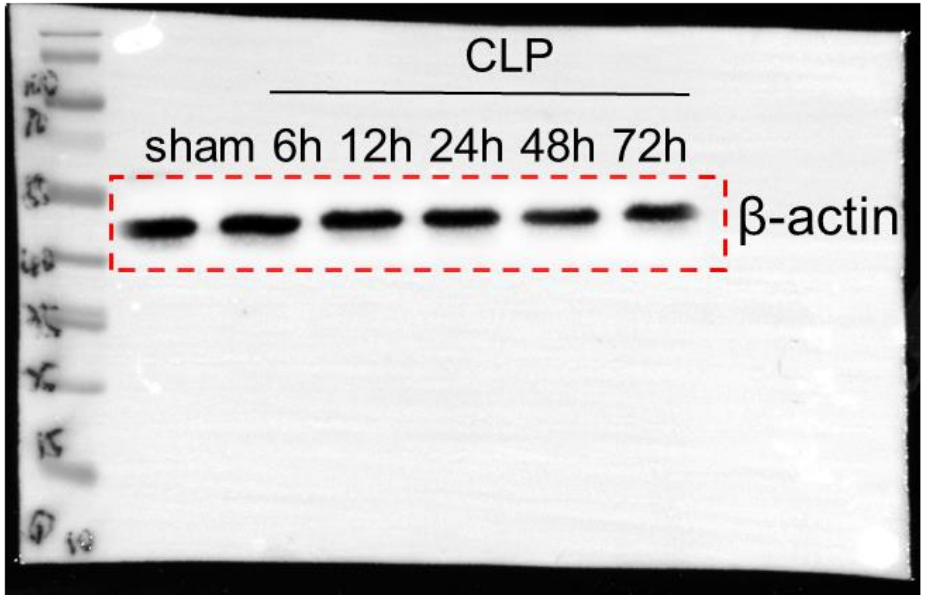

Supplement: Supplementary file 3 [file DataSheet1.pdf]
